# Dynamic genomic architecture of mutualistic cooperation in a wild population of *Mesorhizobium*

**DOI:** 10.1038/s41396-018-0266-y

**Published:** 2018-09-14

**Authors:** Stephanie S. Porter, Joshua Faber-Hammond, Angeliqua P. Montoya, Maren L. Friesen, Cynthia Sackos

**Affiliations:** 1 School of Biological Sciences, Washington State University, Vancouver, WA 98686 USA; 2 Department of Plant Biology, Michigan State University, East Lansing, MI 48824 USA; 3 Department of Plant Pathology, Washington State University, Pullman, WA 99164 USA; 4 Department of Crop and Soil Sciences, Washington State University, Pullman, WA 99164 USA

**Keywords:** Population genetics, Microbial ecology

## Abstract

Research on mutualism seeks to explain how cooperation can be maintained when uncooperative mutants co-occur with cooperative kin. Gains and losses of the gene modules required for cooperation punctuate symbiont phylogenies and drive lifestyle transitions between cooperative symbionts and uncooperative free-living lineages over evolutionary time. Yet whether uncooperative symbionts commonly evolve from within cooperative symbiont populations or from within distantly related lineages with antagonistic or free-living lifestyles (i.e., third-party mutualism exploiters or parasites), remains controversial. We use genomic data to show that genotypes that differ in the presence or absence of large islands of symbiosis genes are common within a single wild recombining population of *Mesorhizobium* symbionts isolated from host tissues and are an important source of standing heritable variation in cooperation in this population. In a focal population of *Mesorhizobium*, uncooperative variants that lack a symbiosis island segregate at 16% frequency in nodules, and genome size and symbiosis gene number are positively correlated with cooperation. This finding contrasts with the genomic architecture of variation in cooperation in other symbiont populations isolated from host tissues in which the islands of genes underlying cooperation are ubiquitous and variation in cooperation is primarily driven by allelic substitution and individual gene gain and loss events. Our study demonstrates that uncooperative mutants within mutualist populations can comprise a significant component of genetic variation in nature, providing biological rationale for models and experiments that seek to explain the maintenance of mutualism in the face of non-cooperators.

## Introduction

Mutualism theory seeks to explain the maintenance of cooperation between species despite the omnipresent threat of selfish, uncooperative mutants [1–6]. Theoretical models of mutualism often assume that cooperation can be lost in a single-mutational step of major effect [7–9]. Empirical model systems also often consider binary shifts in mutualistic cooperation, artificially generating uncooperative mutants that fail to provide host benefit [10–13]. In nature, gains and losses of the genetic modules harboring symbiosis genes punctuate symbiont evolutionary histories and cause transitions between cooperative symbionts and uncooperative free-living lineages, though these are not necessarily antagonistic to their host [14–17]. Some transitions have resulted in spectacular expansion of symbiont or host niches, particularly in symbioses translocated to facilitate the expansion of agriculture [18–21]. A central challenge for understanding the evolution of mutualistic cooperation in natural populations is to understand how frequently such variation in the presence and absence of symbiosis genetic modules impact closely related symbionts competing for host resources.

Empirically, it is controversial as to whether the evolutionary maintenance of mutualistic cooperation, i.e., mutually beneficial interspecific interactions, is threatened by uncooperative genotypes in a tragedy of the commons within a host. This threat would require uncooperative genotypes to gain fitness through their lack of cooperation [6]. Alternatively, uncooperative genotypes may be rare due to host control mechanisms, and thus pose little threat to durable cooperation [5, 6, 22–24]. Furthermore, substantial debate surrounds the question of whether uncooperative genotypes tend to evolve from within a mutualist lineage or whether these potential cheaters tend to comprise distantly related lineages (i.e., third-party mutualism exploiters or parasites [6, 23, 25, 26]). We currently lack a population genomic perspective on the prevalence of the presence and absence of symbiosis genetic modules within populations of close kin, or an understanding of how such major genomic variants impact standing variation in cooperation in natural symbiont populations. If common, such variation would suggest that potential cheaters could evolve easily from within a population of cooperators interacting with a shared host.

In contrast to the binary shifts described above, symbiont cooperation often varies quantitatively [27–29], driven by genetic variation within symbiotic genetic modules [30]. For example, *Ensifer* and *Rhizobium* symbionts possess different complements of accessory genes as well as allelic variants that impact cooperation, often in a manner that depends upon host compatibility [31, 32]. Where cooperation evolves as a quantitative trait, many mutations of small effect underlie standing variation in cooperation [33]. However, because symbiosis genes are often clustered in mobile genetic elements such as integrative and conjugative islands and plasmids [34], cooperation also evolves via mutational steps of major effect whereby lineages lose or gain the entire symbiosis gene modules necessary to engage in mutualism [14, 15, 35, 36]. It is an open question whether these two types of genetic variation underlie evolution on disjunct timescales, or whether they occur simultaneously within a segregating population of symbiotic bacteria.

Co-operation between legumes and rhizobia drives half of all terrestrial nitrogen fixation and is a critical component of sustainable agriculture [37]. In this mutualism rhizobium bacteria acquired by a plant from the soil are housed within root nodule organs, where they fix atmospheric nitrogen in exchange for photosynthetic sugars [38]. Genes that enable cooperation in bacteria often reside on highly mobile genomic elements, which can favor the evolutionary maintenance of cooperation because transfer can increase assortment among alleles for cooperation [39–42]. In *Mesorhizobium*, the genes required for initiating host nodulation, nitrogen fixation, and maintenance of symbiosis are clustered into large single or tripartite symbiosis islands within conserved integration sites in the chromosome (i.e., integrative and conjugative elements [36, 43]). The island can be excised from the chromosome and horizontally transmitted via a type IV secretory apparatus and rolling circle replication into receptive recipient strains [20, 21, 36, 44].

We investigate the genomic basis of variation in cooperation and the pan-genomics of the symbiosis island in a wild *Mesorhizobium* population isolated from root nodules of *Acmispon wrangelianus*, a native plant in California [45]. Draft genome sequences for 48 of these *Mesorhizobium* revealed 38 strains in a focal population are 99.8% identical over a portion of 16S and comprise a recombining population with 97.5% nucleotide identity genome-wide, while ten strains are more distantly related [46]. We focus on the focal recombining population of *Mesorhizobium* microdiversity to ask, (1) How variable is cooperation among *Mesorhizobium* strains that share high genome-wide relatedness? (2) Are there genomic attributes that predict cooperation phenotype among *Mesorhizobium* strains? and, (3) Do genes within the symbiosis island tend to be transmitted in a single tightly linked block or in multiple subclusters?

## Methods

### Genetic variation in cooperation

#### Inoculation experiment

We measured host performance, across three plant genotypes [45], in single-strain inoculations with each of the 38 strains in a recombining *Mesorhizobium* population from [46] (Table S1; Table S2) using a complete factorial randomized block design. The strains and host plants originate from naturally coevolving populations at the Jasper Ridge, McLaughlin, and Hopland Reserves in California, from two natural soil types [46]. These 114 GxG combinations were replicated once in each of two blocks, with uninoculated control plants for each plant genotype, for a total of 256 pots. We removed data from strain NJ11 from analyses involving symbiotic capability because the current stock culture appears contaminated: the *nodA* PCR profile of the stock culture indicates the presence of the symbiosis island (SI), which is not concordant with the absence of the SI in its draft genome [46] (Supplementary Information 1). One-month post-inoculation, plants were harvested for biomass, nitrogen composition, and root nodule estimation. We used PCR and Sanger sequencing to check each strain for *nodA*, which is located on the symbiosis island (Supplementary Information 2).

#### Analysis

To assess rhizobial genetic variation for symbiotic quality, we used a mixed effects general linear model (lme4 [47]), including rhizobium strain and plant genotype as random effects, and soil type, reserve, and block as fixed effects. The interaction between rhizobium strain and plant genotype was not included in the model due to low power to test this term. Significance of random effects was determined with the likelihood ratio statistic. The proportion of total variance explained by both random effects was calculated in analogous models fit by restricted maximum likelihood. Assumptions of normality and homogeneity of variance were assessed graphically [48].

### Genomic attributes predicting cooperation and pan-genomics of the SI

#### Symbiosis genes and genomic attributes predicting cooperation

We defined high-confidence SI genes that both: (1) impact symbiosis and nitrogen fixation based on a comparative study of *Mesorhizobium* (106 genes [44]), or impact horizontal gene transfer of the SI (44 genes with 41 unique accessions [43]), and (2) map to the SI in the reference genome of *Mesorhizobium loti* strain MAFF303099 (601 genes [49]). This list is conservative rather than exhaustive and contains many loci with well-established impacts on symbiosis. We used one-way ANOVAs (lm [50]) to examine relationships between the level of host cooperation, the number of symbiosis genes, and the total size of a draft genome.

#### Comparative genomic analysis

The bimodal distribution of the number of SI genes among the 38 strains delineates two bioinformatic categories within the recombining focal population: (1) 32 strains with the SI (*focal_SI+*) and (2) six strains without the SI (*focal_SI-*). We also consider ten non-focal population strains with the SI (*nonfocal_SI+*). We used LASTZ [51] alignments to calculate average nucleotide identity (*ANI*) for full genomic alignments. To identify genomic regions associated with symbiosis that have undergone horizontal gene transfer (HGT), we identified conserved sequence blocks among divergent pairs of *focal_SI+* and *nonfocal SI+* strains, then removed those that were found in alignments between any *focal_SI+* and any *focal_SI−* strain (Supplementary Information 1; https://github.com/jfaberha/lastz_lav_expansion). Candidate HGT symbiosis genes were clustered based on presence/absence with Ward.D2 clustering [52] and Euclidean distance method. This allowed us to distinguish clusters of HGT genes with highly variable presence/absence patterns from those likely coinherited. We define genes present in nearly all SI+ strains based on clustering profiles as “near-core” SI genes. Bootstrap values for gene clusters were computed using 1000 iterations in pvclust [53]. We tested for functional gene ontology (GO) enrichment for the 177 genes in the putative SI and 1186 rare HGT genes (Blast2GO v3.2 [54] as compared to the remaining genes in the *Mesorhizobium* pan-genome defined by *M. loti* strain MAFF303099 [49] plus de novo genes annotated in the focal *Mesorhizobium* population in [46] at FDR < 0.05.

#### Impact of SI on plant fitness

We used a one-way ANOVA (lm [50]) to test whether the presence of the SI is related to a strain’s level of cooperation. We included host genotype and block in all models and analyzed both shoot mass (log-transformed) and percent nitrogen in leaf tissue. We further tested whether the *focal_SI−* strains differed from the uninoculated control treatment using a general linear model on this data subset (lm [50]) and corrected for multiple tests using the Sequential Bonferroni procedure [55].

Within the *focal_SI+* strains, we took a GWAS approach to test for associations between the presence/absence of individual genes and cooperation phenotype using a Wilcoxon rank sum test with false discovery rate correction using the analyses, SNPs, and gene presence/absence data presented in [46], except that we did not account for population structure as it is heterogeneous in the island.

### Patterns of relatedness for the SI

To determine patterns of relatedness among strains for horizontally transferred genes, we calculated *ANI* for each core non-SI gene and each near-core SI using ClustalO distance matrices weighted by the alignment length (Supplementary Information 1). To determine coinheritance, we clustered genes based on pairwise sequence similarities for each set of orthologs among all SI+ strains using Ward.D2 clustering and Euclidean distance methods (as above), with bootstrap values generated with 1000 iterations (pvclust [53]). We also generated unrooted maximum likelihood trees for each near-core HGT gene using a GTRGAMMA model (RAxML v8.2.10 [56]) and ran pairwise tree topology comparisons for all 177 genes (TOPD v4.6 [57]). We calculated split distance, an index based on the number of shared partitions between trees, and nodal distance, a metric of path-length between node placement on each tree [58, 59]. We summarized split and nodal distances for genes within and between significant gene clusters by ranking split and nodal distances for all tree comparisons, then used non-parametric one-way Mann–Whitney *U* tests [60] to check whether within-cluster tree comparisons differ from between-cluster comparisons. To compare phylogenetic profiles of core non-SI and SI genes we built neighbor networks for: (1) 100 random core non-SI genes and (2) concatenated clusters of near-core SI genes using SplitsTree [61].

### Stability of the SI under laboratory conditions

To investigate whether the SI is lost under experimental culture conditions, we grew eight SI+ strains through four serial transfer events over 1 month of continuous liquid culture, then conducted colony PCR for partial *nodA* and partial 16S loci. A subset of PCR products were Sanger sequenced (Supplemental Information 1 & 2).

## Results

### Abundant genetic variation in cooperation

**Fig. 1:**
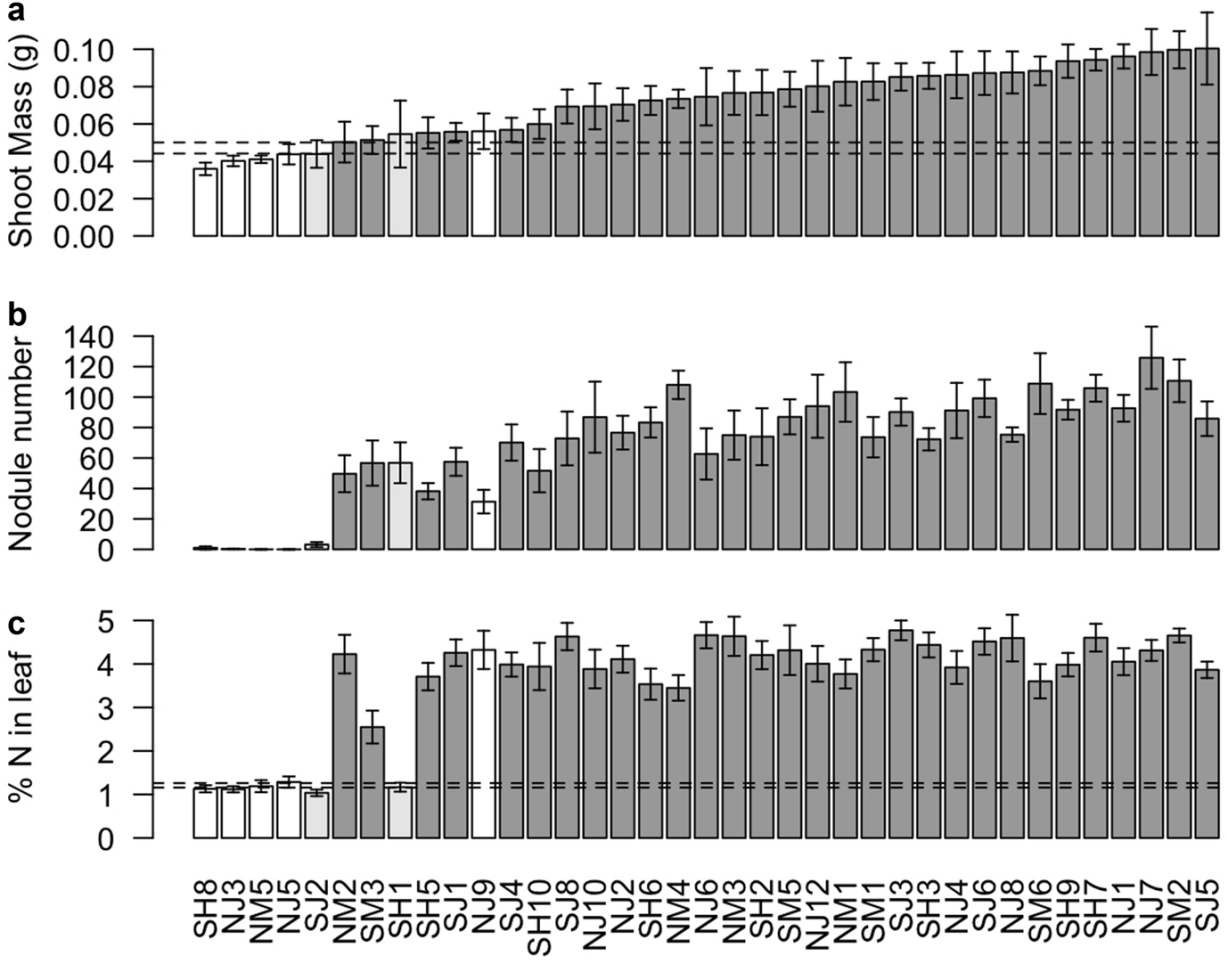
Abundant variation in cooperation among closely related strains of *Mesorhizobium*. Shown are shoot biomass (**a**), root nodule number (**b**), and percent of leaf tissue comprised of nitrogen (**c**) for the host plant, *Acmispon wrangelianus*, when inoculated with different strains. Bars indicate mean *Mesorhizobium* genotype effects averaged across three coevolved, inbred plant lines (*n* = 234 pots). Genomic analysis reveals three categories of strains: strains that lack the symbiosis island (white), strains that contain an intermediate number of symbiosis genes (light gray), and strains that contain nearly all symbiosis genes as well as the full symbiosis island (dark gray). Error bars indicate standard error. Dashed lines indicate standard error around the means for *Mesorhizobium*-free plants. *Mesorhizobium*-free plants did not form nodules

Closely related strains show abundant symbiont genetic variation for shoot mass conferred to a host (*χ*^2^ = 10.973, *P* = 0.0009), nodulation (*χ*^2^ = 92.967, *P* < 2.2e−16), and percent nitrogen composition in leaf tissue (*χ*^2^ = 157.77, *P* < 2.2e−16) (Fig. 1). After accounting for fixed effects (soil type, reserve, and block; none of which were significant), rhizobium genotype explains a large proportion of the total variance in these response variables (Table 1).

**Table 1:** Sources of variance in mixed models of plant trait values

Random effects	Shoot mass	Nodule number	Percent nitrogen in leaf
Symbiont genotype	31%	50%	64%
Host genotype	7%	10%	6%
*Residual*	*62%*	*40%*	*30%*

Traits were expressed in the greenhouse by factorial combinations of three *Acmispon wrangelianus* lineages and 37 *Mesorhizobium* strains. Presented are variance components for random effects and residual variance not accounted for by predictors as percentages of total phenotypic variance, after accounting for the fixed effect of soil type, reserve, and block.

### Genomic attributes predicting cooperation and pan-genomics of the SI

#### Genome analysis

**Fig. 2:**
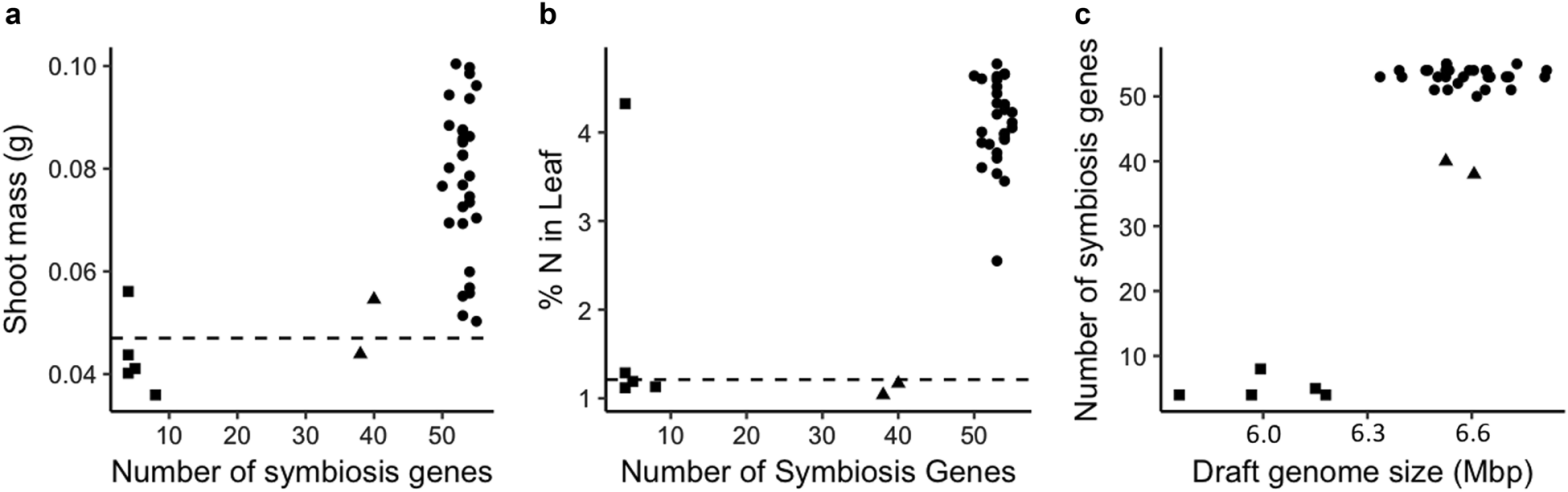
*Mesorhizobium* strains isolated from nodules tend to be less cooperative if they contain fewer symbiosis genes. Strains with fewer symbiosis genes confer less shoot mass (**a**) and nitrogen (**b**) to *Acmispon wrangelianus*. Strains with smaller draft genomes also contain fewer symbiosis genes (**c**). Genomes that contain a majority of our high-confidence symbiosis genes (circles) are on average 583 Kbp larger than those with few (squares), which is the approximate size of the *Mesorhizobium* symbiosis island. Two strains have an intermediate number of symbiosis genes (triangles). Dashed lines indicate the level of a plant trait expressed by uninoculated control plants

**Fig. 3:**
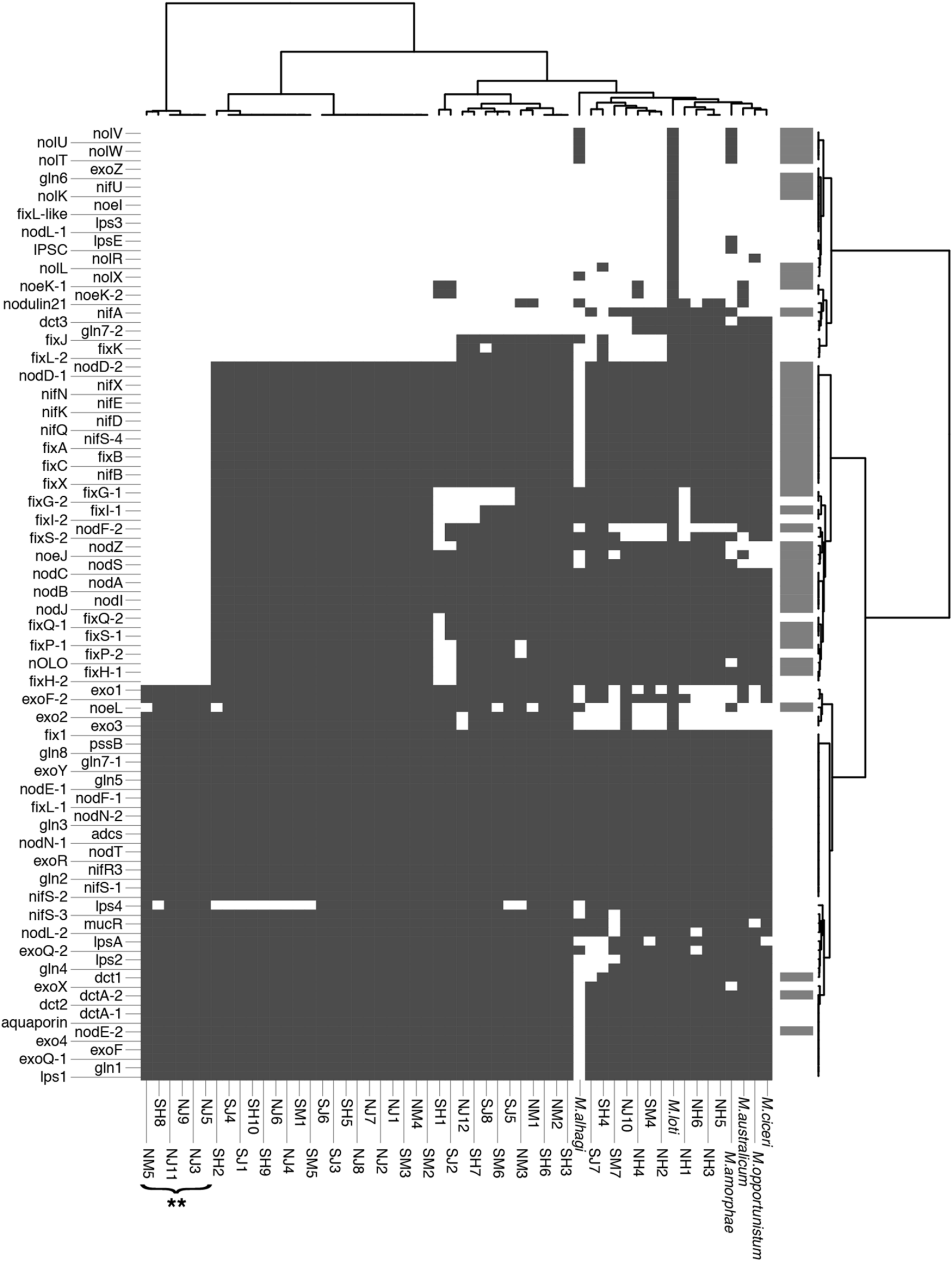
Presence/absence of 104 genes associated with symbiosis and nitrogen fixation across wild *Mesorhizobium* and published reference strains. Gene list from ref. [44]. Dark gray indicates a gene is present in a strain. For the reference strain, *Mesorhizobium loti* (MAFF303099), the subset of loci present in the symbiosis island are indicated in light gray and encompass the high-confidence symbiosis genes. **Strains from the focal population that lack the symbiosis island (*focal*_*SI-*)

**Fig. 4:**
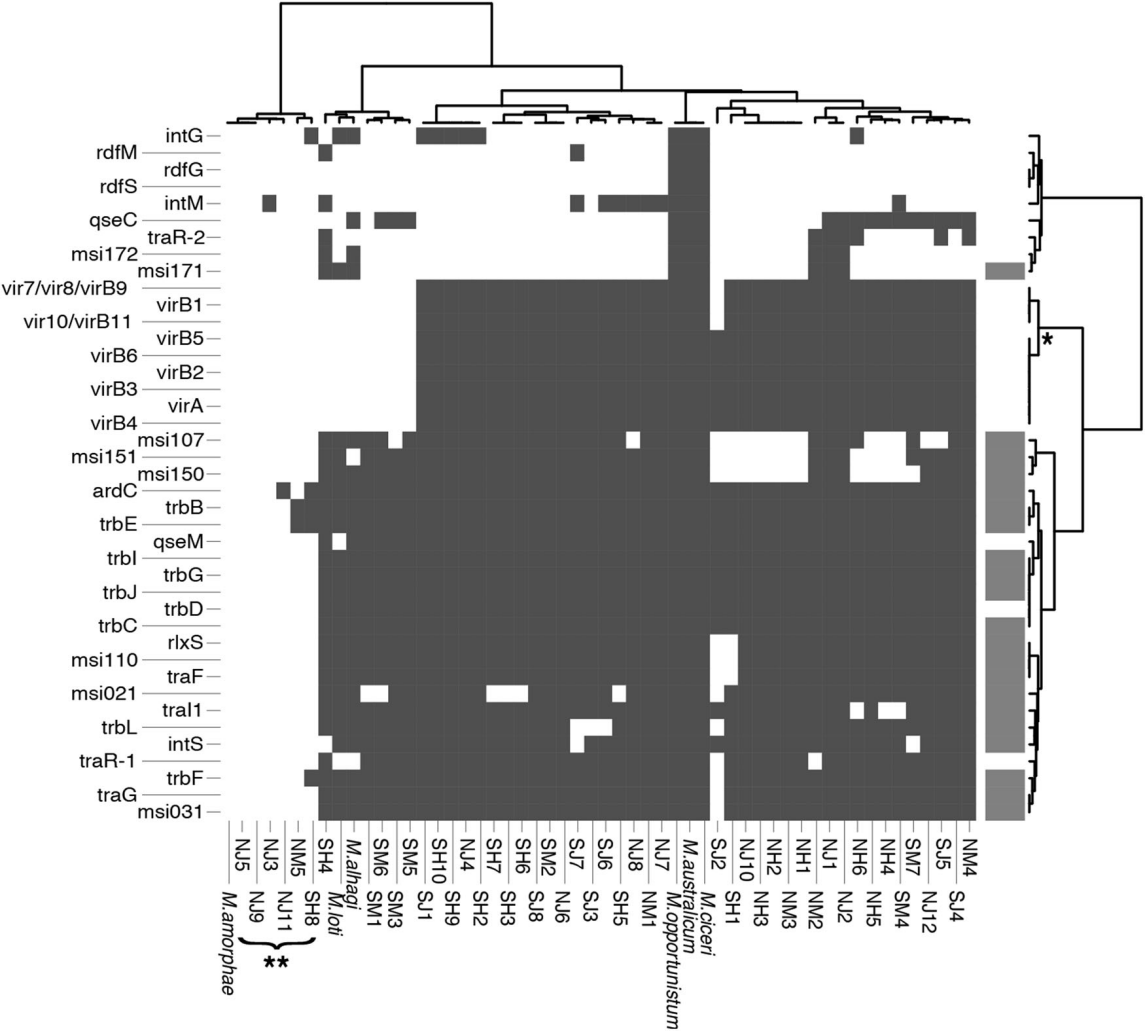
Presence/absence of 41 genes involved in horizontal gene transfer of the *Mesorhizobium* symbiosis island. Gene list from [43]. Dark gray indicates a gene is present in a strain. For the reference strain, *Mesorhizobium loti* (MAFF303099), the subset of loci present in the symbiosis island are indicated in light gray. *Cluster of genes with a unique presence/absence profile; **Strains from the focal population that lack the symbiosis island (*focal*_*SI-*)

Of the 66 high-confidence symbiosis genes shared between three lists of symbiosis-related genes, no more than 55 are found in any strains. We find a strong bimodal distribution of high-confidence symbiosis genes among strains (Fig. 2), reflecting presence/absence of the SI (Figs. 3 and 4). Of the 48 strains, 42 contain 38–55 of the 55 high-confidence SI genes, while 6 strains have only 4–8 of these genes. Four predicted symbiosis-related genes are present in *focal_SI−* strains: *nodulation protein nodE* (GI:13474847), *C4-dicarboxylate transport system regulatory protein* (GI:13474866), *GDP-D-mannose dehydratase*/*nodulation protein noeL* (GI:13474933), and *C4-dicarboxylate transporter dctA* (GI:161621446). Four SI HGT-associated genes are present in some SI*−* strains: *ardC* (GI:13475143), *trbB* (GI:13475352), *trbE* (GI:13475354), and *trbF* (GI:13475357). It is possible the loci we identify are paralogs of symbiosis loci.

#### Comparative genomic analysis

Based upon bioinformatic data, 6/38 strains from the focal population appear to lack the SI (15.7%), and 0/10 of the strains from outside of the focal population appear to lack the SI. Several lines of evidence support this delineation. First, high-confidence SI genes lie in close physical proximity within contigs. In addition, in concordance with the known architecture of the *Mesorhizobium* SI, one major cluster of SI genes resides ~300 Bp upstream of the SI integration site phe-tRNA [18], with *SI integrase* (*intS*) right at this boundary. The *intS* insertion points *attL-S* and *attR-S* [43] are located ~200 Bp upstream of *intS*. These reverse complementary *att* sequences are 17 bp long and found in full in few other loci in our draft genomes, although in these cases paralogous *att* loci are not adjacent to any known SI sequence. Non-SI contigs containing *att* loci often contain *ABC transporter ATP-binding protein*, *glycosyl transferase*, and *type I secretion system abc family* genes, and are likely associated with separate HGT events. Other SI-associated integrases, *intM* and *intG*, are rare in our strains and while their associated *attL* and *attR* sequences are found within 350 and 150 bp of the genes, respectively, these short sequences map to dozens of loci throughout the genome and were not detected as HGT content by our pipeline. In addition, the majority of high-confidence SI genes are present in SI+ strains but absent in SI*−* strains (Figs. 3 and  4) and fall within two main contigs in each genome with small clusters in additional contigs, although these regions show high internal variability in genomic architecture. Furthermore, the number of high-confidence symbiosis genes correlates with draft genome size, which suggests that the bimodal distribution of genome size is driven by the presence/absence of the SI. The average difference between SI+ and SI*−* genomes is 582.95 Kbp, with an average genome size of 6.58 Mbp and 6 Mpb, respectively (Fig. 2c).

#### Delineation of the SI

A total of 1363 unique genes are detected as horizontally transferred between *focal*_*SI+* and *nonfocal*_*SI+* strains. Clustering of HGT gene presence/absence patterns reveals a natural break between near-core genes present in nearly all SI+ strains and rare genes with variable presence/absence patterns (bootstrap value, 74) (Fig S1). We infer 177 near-core HGT genes co-segregate as the SI, while latter group is comprised of 1186 genes that either independently horizontally transferred or are rare hitchhiking genes integrated into the SI. As evidence, the set of near-core genes contains many known *Mesorhizobium* SI genes from published genomes, and the rare HGT genes are largely annotated as transposon related, and physical gene mapping suggests many of these are integrated into the SI in certain strains (Table S3). The genomic intervals encompassing the near-core HGT genes in SI+ strains have an average length of 444.95 Kbp similar to the 502 Kbp SI in *M. japonicum* R7A (previously classified as *M. loti*) [62] and the 611 Kbp SI in *M. loti* MAFF303099 [49].

GO enrichment tests on both the near-core HGT gene set and rare HGT gene set yield unique sets of enriched GO IDs (reduced to most specific terms; FDR < 0.05), which suggests the sets are biologically distinct (Table S4). The near-core gene set is primarily enriched in symbiosis and nitrogen fixation-related GO terms. We identified 33 symbiosis genes based on GO classification (GO:0044403) that do not appear to reside within the SI in wild strains, eight of which are present in *focal_SI−* strains, including apparent duplicates of *NodE*, *NodF*, and *NodG*, which suggests they are integrated into the main chromosome (Table S5).

#### Genomic attributes predicting cooperation

The number of symbiosis genes (Fig. 2a, b) and the total size of the draft genome for a strain are both strong predictors of host shoot mass (*F*_1,35_ = 25.16, *P* = 1.53 × 10^−5^; *F*_1,35_ = 6.77, *P* = 0.013, respectively) and percent nitrogen in leaf tissue (*F*_1,35_ = 35.84, *P* = 8.05 × 10^−7^; *F*_1,35_ = 11.89, *P* = 0.0015, respectively). After excluding SI− strains, this relationship remains significant for number of symbiosis genes (Shoot mass: *F*_1,30_ = 4.757, *P* = 0.03715; percent nitrogen in leaf: *F*_1,30_ = 53.11, *P* = 4.09 × 10^−8^), but not for draft genome size. The number of symbiosis genes in a draft genome is a strong predictor of total draft genome size (*F*_1,35_ = 85.22, *P* = 6.58 × 10^−11^; Fig. 2c), though this relationship is not significant among SI+ strains (*F*_1,35_ = 0.0055, *P* = 0.94).

The inferred presence or absence of the SI is a strong predictor of the shoot mass and leaf N content attained by inoculated host plants (*F*_1,35_ = 20.01, *P* = 7.79 × 10^−5^; *F*_1,35_ = 21.63, *P* = 4.59 × 10^−5^, respectively; Fig. 2a, b). While some of the five SI− strains for which we estimated symbiotic effects trend toward negatively impacting host shoot mass as compared to uninoculated plants (Fig. 1), once contrasts between these values are corrected for five multiple tests, the impact of individual SI− strains on shoot mass are indistinguishable from that of the uninoculated plants (Supplemental Information 3).

Within the set of SI+ strains, no individual SI genes have SNPs or gene presence/absence patterns that are associated with shoot mass conferred to a host, even with a lenient FDR < 0.20 threshold. We identify ten genes outside of the SI with presence/absence patterns associated with shoot mass conferred to a host at FDR < 0.10, and two additional genes with FDR < 0.20 (Table S6).

### Patterns of relatedness for the SI

**Fig. 5:**
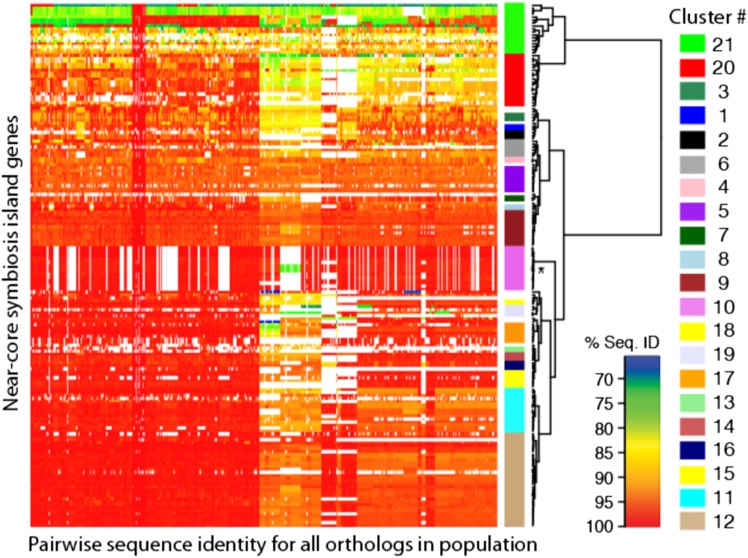
Heat map comparison of pairwise sequence identity for 177 symbiosis genes across 42 wild *Mesorhizobium* strains shows 21 clusters of symbiosis genes with contrasting patterns of relatedness among strains. The *x*-axis indicates all 861 pairwise comparisons for each gene between 42 SI+ strains. The *y*-axis indicates 177 near-core symbiosis island genes. Colored bars to the right of the heat map indicate Ward clusters of genes with bootstrap values ≥ 90. *Cluster 10 contains *vir* genes which are implicated in the horizontal transfer of the symbiosis island and are found in SI+ but not SI*−* wild strains but are not contained in the *Mesorhizobium loti* MAFF303099 symbiosis island. See Fig. S2 for gene names along the *y*-axis

Within *focal*_*SI+* strains, orthologous near-core and core SI genes share *ANI* of 92.0% and 97.6%, respectively, while they share *ANI* of 87.6% and 97.6% between *focal*_*SI+* strains and *nonfocal_SI+* strains. To examine substructure and possible coinheritance within the near-core SI genes, we clustered genes by pairwise sequence similarity among orthologs. This reveals a variety of coinheritance patterns (Fig. 5, Fig S2), with 21 significant gene clusters based on coinheritance profiles. Genes within clusters are often directly adjacent to each other within assembled contigs (Table S3) and share fine scale patterns of gene presence/absence among strains, suggesting that clustered genes are acquired or lost as a unit (Table S7).

**Fig. 6:**
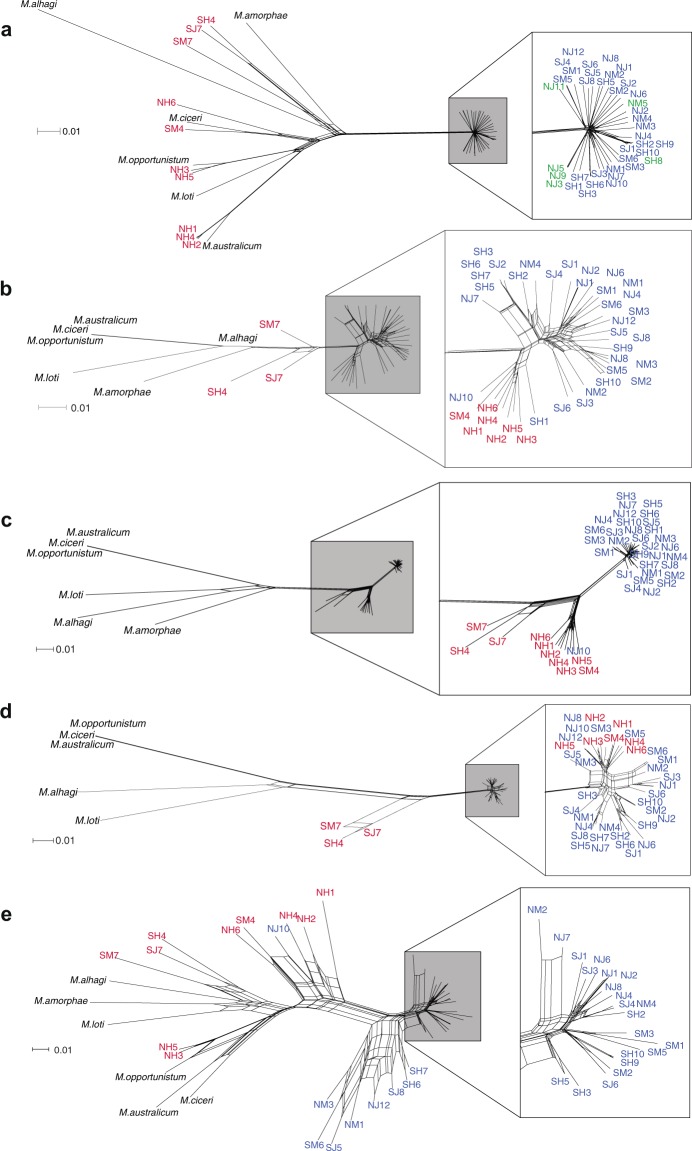
Neighbor networks highlight differences in relatedness patterns between strains based on genomic sequence and several symbiosis island gene clusters. Networks for (**a**) 100 random core single-copy genomic genes, and for the following near-core symbiosis island gene clusters: (**b**) cluster 9 (12 genes), (**c**) cluster 12 (32 genes), (**d**) cluster 15 (6 genes), and (**e**) cluster 20 (18 genes). Strain coloration: blue, *focal_SI+* wild *Mesorhizobium* population; green, *focal_SI−* wild *Mesorhizobium* population (**a** only); red, sympatric *non-focal_SI+* population strains; black, publicly available *Mesorhizobium* outgroups

Neighbor Networks for concatenated sequence alignments of significant near-core SI gene clusters with >5 genes show different patterns of relatedness among strains than those based on core non-SI genes (Fig. 6). Wild strains are closely related relative to fully sequenced *Mesorhizobium* outgroups for gene clusters 11, 12, and 17 (Fig. 5). Strains show long branch lengths for clusters 5, 9, and 21 (Fig. 5), possibly indicating a lack of recent HGT between strains or a high substitution rate. *Focal*_*SI+* and *non-focal*_*SI+* strains are closely related and have short branch lengths relative to publicly available outgroups for clusters 10 and 15, possibly due to recent and sustained HGT or conservation among wild strains. Cluster 10 genes do not occur in the *M. loti* MAFF303099 SI, but they have been implicated in SI HGT [43] and for these genes, wild strains show a presence/absence pattern distinct from that of other near-core SI genes. Comparisons of ML phylogenetic tree topologies among SI gene clusters using non-parametric Mann–Whitney *U* tests support these results and show that within-cluster topologies are more similar than between-cluster topologies using metrics for both split distance (*W* = 9,225,323; *P* = 1.20e−54) and nodal distance (*W* = 8,061,162; *P* = 7.20e−11 for pruned trees, *W* = 8,769,688; *P* = 3.02e−33 for unpruned trees). Functional analysis of large gene clusters indicates most are enriched for symbiosis and/or nitrogen fixation GO terms, although there is some variation in specific terms suggesting the possibility of SI organization into distinct functional operons (Table S4).

### Stability of the SI under laboratory conditions

PCR and sequence data indicate that the symbiosis island is retained in our wild *Mesorhizobium* strains despite passage through serial cultures in the laboratory. Electrophoresis of *nodA* PCR products suggest none of the eight SI positive strains lost the SI after four weeks of serial transfer in liquid culture (~45–170 generations). Sanger sequencing of a subsample of these PCR products confirmed them to be the target *nodA* and *16S* sequences.

## Discussion

Research on mutualisms seeks to explain how cooperation can be maintained when uncooperative mutants co-occur with cooperative kin [1–6, 63–66]. We provide genomic evidence that variation in the presence or absence of the large (~500 Kbp) island that confers the ability to cooperate with a host are common within a single wild recombining population of symbiotic microbes. In the focal *Mesorhizobium* population we examine, uncooperative variants that lack a symbiosis island segregate at ~16% frequency. We designate these strains as uncooperative because although they were isolated from root nodules, and thus were symbiotic with a host plant, they do not provide services to their host and thus cannot cooperate [30, 63]. Uncooperative rhizobium lineages that lack symbiosis gene islands or symbiosis plasmids are common in *Bradyrhizobium* [14–16, 67, 68], *Rhizobium* [69, 70], and *Mesorhzobium* [19]. However, we know of no other genomic evidence that strains from a single recombining wild population of symbionts isolated from host modules differ primarily in the presence and absence of the genomic island encoding the ability to cooperate. Our findings are concordant with findings in free-living bacteria in which variation among strains in the presence and absence of cooperative traits residing on mobile elements is common [39–42]. We provide clear evidence that models and experiments seeking to explain the evolution of mutualist cooperation in the face of uncooperative mutants that evolve from within mutualist populations address a fundamentally important source of genetic variation in nature. This finding contrasts with the genomic architecture of variation in mutualistic cooperation in other symbiont populations isolated from host tissues in which the islands of genes underlying cooperation are ubiquitous and variation in cooperation is primarily driven by allelic substitution and individual gene gain and loss events [31, 71–74].

### Genomic attributes predicting cooperation

Despite high genome-wide relatedness among mesorhizobia in the focal population, these strains exhibit dramatic variation in cooperation. Much of this variation can be predicted by genome attributes: strains with few symbiosis genes and strains with smaller genomes tend to be less cooperative. Even after excluding strains lacking the symbiosis island, strains in the focal population with fewer symbiosis genes tend to be less cooperative. In contrast to our findings, small genome size is correlated with greater benefits to a host among symbiotic bacteria in vertically transmitted symbioses [75]. Furthermore, among diverse symbioses involving horizontally transmitted bacteria, there is no relationship between bacterial genome size and the level of cooperative benefits a host receives [76]. The positive correlations between genome size, symbiosis gene number, and cooperation we find at the population level highlights the potential to use these attributes to predict symbiont quality at the population level if the genomic architecture of cooperation is labile.

The distribution of symbiosis genes, genome size variation, and measures of cooperation partition our 48 wild strains into those with or those without the symbiosis island. Our wild mesorhizobia harbor 55 high-confidence symbiosis island genes that are bimodally distributed among strains: 42 strains with large genomes contain the vast majority of these genes, while 6 strains with small genomes contain only a handful. Furthermore, the genome sizes of strains with many vs. few high-confidence symbiosis island genes are non-overlapping: the 42 strains with many symbiosis island genes have an average total assembly size of 6.58 Mbp, (range: 6.34–7.79 Mbp), while the six small draft genomes with few symbiosis island genes average 6.00 Mbp (range: 5.75–6.18 Mbp). Notably the difference between these averages, 580 Kbp, is close to the size of symbiosis islands in fully sequenced *Mesorhizobium* genomes [49, 62]. Non-nodulating rhizobia that lack symbiosis islands or plasmids can suppress nodulation of host legumes by cooperative strains [77, 78], and diverse non-rhizobium microbiota regularly colonize healthy nodule tissue [79, 80]. In wild bradyrhizobia, uncooperative strains can co-found nodules with cooperative strains where they can have negative impacts on the fitness of both the host plant and the strain with which they co-invade [67].

In the recombining *Mesorhizobium* population we study, strains that differ in the presence or absence of the entire symbiosis island are common in host nodules, and this stands in stark contrast to the population genomics of symbiosis plasmids in other rhizobial populations. Draft genome sequencing of some wild symbiotic *Ensifer* and *Rhizobium* populations indicates their symbiosis replicons evolve via allelic substitution and the gain and loss of individual genes, rather than the gain and loss of entire symbiosis islands or replicons [31, 71, 72, 81]. In *Mesorhizobium*, symbiosis genes tend to be grouped together in integrative and conjugative elements (i.e., symbiosis islands) that can be readily horizontally transferred or lost and gained [18, 19, 43], whereas in the Rhizobiacea genus *Ensifer*, symbiosis genes reside on symbiosis megaplasmids that are difficult to eliminate, even with targeted molecular genetic approaches [82]. While absence of the symbiosis island is common in the *Mesorhizobium* we study, losses of the island may be rare over ecological time, as we observed no losses of the island during one month of serial transfers in liquid culture. It is possible such losses could be more common in plant tissues [83]. Differences in the stability of genomic islands or plasmids among symbiont clades likely result in phylogenetically predictable patterns of microbial genome evolution in populations of these symbionts, which could underlie contrasting patterns of co-evolutionary selection. Future research could reveal whether the frequency of non-cooperative mutants differs among rhizobial symbiont genera and whether any such differences drive different co-evolutionary dynamics with host populations, as may occur for symbiotic pollinating wasp genera on host fig trees [30].

In addition to the clear effect of the symbiosis island on cooperation, we also detect a small number of genes not present on the island that are correlated with cooperation. Although the symbiosis island varied in both content and recent evolutionary history among strains, genes from the symbiosis island are not statistically associated with cooperation phenotype. Of the 10 genes from outside the island that did associate with cooperation, two are oligopeptide ABC-transporters, which have been previously shown to be involved in both symbiosis and stress tolerance [84].

Uncooperative SI+ or SI*−* strains could both pose a challenge to the maintenance of mutualism but differ in one important respect. Uncooperative SI+ *Mesorhizobium* are able to found nodules, and if these uncooperative strains are unchecked by host control mechanisms like partner choice [5, 85], nodules founded by uncooperative SI+ strains could increase in frequency, displacing more cooperative SI+ strains in the symbiotic population, and lead to complete mutualism breakdown [27, 86]. In contrast, uncooperative SI− strains do not generally appear to found nodules on their own. While SI*−* strains were isolated from nodules, we hypothesize that they may co-infect nodules that are founded by SI+ strains. Non-nodulationg strains commonly co-infect nodules with nodulating strains in *Acmispon strigosus* [67], a close relative of *A. wrangelianus*. If uncooperative SI*−* strains become more common, frequency dependent selection against them will increase because cooperative SI+ strains will become rare [87]. If however, SI*−* strains maintain high fitness in the free-living state [15, 16], perhaps benefiting from alleviation of costs associated with the 500 Kbp SI, they may parasitize cooperative strains that do nodulate, and thus threaten the maintenance of cooperation in their SI + kin.

In our study, strains lacking the island tend to be non-nodulating and confer no benefit to the host in single-strain inoculations, while strains possessing the island induce nodules and tend to confer host benefit. However, there was one notable exception: in some cases, strain NJ9 nodulates at low levels, despite the fact that it lacks the island. Strain NJ9 forms the fewest nodules among the strains capable of forming nodules, and these nodules likely indicate a low frequency strain containing the island contaminant in the NJ9 culture, which could remain undetected by PCR of *nodA* and in the draft genome analysis yet could result in nodulation under conditions of high density inoculum [67].

### Pan-genomics of the symbiosis island

Our findings support the emerging perspective of rhizobium symbiosis islands and plasmids as hotspots of horizontal transfer, recombination, and gain and loss events [31, 70–72, 82, 88, 89]. While genome-wide patterns of relatedness indicate that our focal mesorhizobia comprise a recombining population [46], patterns of relatedness among the symbiosis islands of these strains indicate complex population genetic subdivision marked by recombination. Thus, as is common in rhizobia, the evolutionary history of the symbiosis island contrasts with the evolutionary history of the main chromosome in which the symbiosis island is integrated [16, 71, 72, 89, 90]. Future work could establish how such incongruence impacts cooperation.

The symbiosis island appears comprised of a mosaic of distinct functional units/operons subject to distinct patterns of transmission, as previously observed in pangenomic analyses of rhizobium populations [72, 74, 81]. We identify 1363 genes with distributional patterns that suggest they have been horizontally transferred among wild strains. In total, 177 of these genes are present in almost all strains and this set is highly enriched for “symbiosis” and “nitrogen metabolism” GO categories; we infer this set to comprise the putative symbiosis island. Hierarchical clustering based on pairwise-relatedness reveals substantial substructure among 21 distinct clusters of genes in the symbiosis island. Genes within these clusters tend to be adjacent to one another in genomic contigs and show different Neighbor Net patterns of relatedness among strains, suggesting that these gene clusters have contrasting evolutionary histories. Because our draft genomes are incomplete, we cannot comprehensively determine physical linkage. It is possible that different phylogenetic signals within the island are driven by variable rates of recombination among different segments. A tripartite system of symbiosis island HGT occurs in some *Mesorhizobium* [43, 91]. It is possible that our wild strains use an analogous mechanism of transfer of multiple or individual island fragments.

Genes that underlie nodulation and symbiotic nitrogen fixation are widely distributed across these clusters of near-core symbiosis genes. The largest cluster of near-core genes, Cluster 12, contains 32 genes and includes a wide variety of *nod*, *nif*, and *fix* genes, which are integral to symbiosis with the plant host [44]. Many key symbiosis genes reside in clusters with different phylogenetic profiles. For example, Cluster 20 contains of a suite of multi-copy *fix* genes (*fixG-I*, *fixP*, and *fixS*) and Cluster 5 contains several *nif* genes (*nifE*, *nifK*, and *nifN*). These distinct clusters therefore both encode components of the nitrogenase enzyme and associated proteins responsible for nitrogen fixation [44]. Cluster 9 contains *nodD*, which is involved in perception of host flavonoid signals [92], and *nodO*, which impacts nodule formation [93]. Notably, symbiosis genes often used as phylogenetic markers for the island such as *nifD, nodC*, and *nodD* [94, 95] fall within different clusters and patterns of relatedness among strains at these individual loci do not appear to be representative of the symbiosis island overall.

We identify symbiosis genes with unexpected presence/absence patterns in the focal populations. First, we detect apparent symbiosis genes that reside outside of the symbiosis island. Strains lacking the island harbored appear to harbor eight high-confidence symbiosis genes, and an additional eight genes from the *Mesorhizobium* pan-genome with the GO annotation, “symbiosis, encompassing mutualism through parasitism” (GO:0044403). It is possible that these genes could play a role in infection of strains lacking the island into host nodules under conditions in the wild, though they could also be paralogs that encode alternative functions. Another set of island genes has a unique presence/absence pattern: genes from near-core cluster 10 are consistently missing in five strains that possess the island. This cluster contains multiple *vir* genes, which encode components of the conjugative Type IV secretion system by which a single-stranded copy of excised and circularized symbiosis island is transferred to a recipient genome [43, 91]. Future studies using finished genomes will be important to map the physical location of the island and these associated genes.

## Conclusions

Variation in the presence or absence of a large island of genes that confer the ability to cooperate with a host appears to be common within a wild recombining population of microbial symbionts. If cooperative and non-cooperative strains co-found nodules in nature, cooperative strains that invest in nitrogen fixation could be competing directly with uncooperative kin that are genetically incapable of fixing nitrogen. In future co-infection experiments we hope to elucidate the selection these strains may exert on other participants in the mutualism [6, 96, 97], to determine whether the *Mesorhizobium* lacking the symbiosis island we observe are cheaters that free-ride on cooperative kin or hosts. Alternatively, when they co-infect nodules with cooperators, uncooperative strains could be selected against through host sanctions operating within nodules [98, 99]. In this case, their prevalence in the population would require other explanations such as tradeoffs between symbiotic capabilities and performance in the free-living state [100] since we do not observe a high mutation rate to this state. This population represents a unique natural mutualism wherein variation in the presence or absence of the genomic capacity to be cooperative and uncooperative occur within a single recombining population of close kin–precisely the mutants considered in many theoretical and empirical studies of mutualism [1–13].

## Electronic supplementary material

Supplementary Information 1

Supplementary Information 2

Supplementary Information 3

Supplementary Information Legend

Figure S1

Figure S2

Table S1

Table S2

Table S3

Table S4

Table S5

Table S6

Table S7
