# Supplementary material for: Dynamic genomic architecture of mutualistic cooperation in a wild population of Mesorhizobium
Source: ISME J. 2018 Sep 14;13(2):301–15. doi: 10.1038/s41396-018-0266-y (PMC6331556; doi:10.1038/s41396-018-0266-y)
Supplement: Supplementary file 1 — Supplementary Information 1 [file 41396_2018_266_MOESM1_ESM.docx]

**Supplementary Information 1**

**Detailed Methods**

***I. Quantitative variation in cooperation***

*Inoculation experiment*. We measured plant fitness in symbiosis with each of the 38 strains in a recombining population of wild Californian *Mesorhizobium* strains described in (1)*.* The three plant genotypes inoculated with these strains were collected in a previous study from each of the three natural reserves from which the strains originate (2). Plant genotypes used were: SJ1C11, NM43H2, SH1E11, originating from the Jasper Ridge, McLaughlin, or Hopland Reserves, respectively. The 38 strains from (1) originated from two contrasting soil types and from three natural reserves. To minimize the impacts of maternal effects, we created three inbred lines by allowing three field-collected seeds to self-fertilize for a generation in a common greenhouse environment. Each strain was singly inoculated onto each host genotype. The resulting 114 GxG combinations were replicated once in each of two complete randomized blocks. Four to five replicates of each host genotype were inoculated with rhizobium-free sterile water in each block and none of these (n=28) formed nodules. There were 256 pots total. We removed data from strain NJ11 from all analyses involving symbiotic capability. The stock culture of this strain appears to be contaminated because the *nodA* PCR profile of the stock culture is not concordant with *nodA* in its draft genome (42).

Seeds were scarified using fine-grit sandpaper, surface-sterilized with chlorine gas for six hours, and vernalized without imbibing the seeds, in darkness at 4^o^C for seven days. Dry seeds were planted into 66 mL cylindrical pots (RLC4, Stewe & Sons, Inc.) containing an autoclaved 1:1 mix of potting soil (Professional Grow Mix, Sungro Horticulture) and inert quartz sand on October 21st, 2016, in the Washington State University, Vancouver greenhouse (45.732674, -122.635735). To simulate the early spring conditions under which *A. wrangelianus* typically establishes, the greenhouse was maintained with 12 hour, 10^o^C nights, and 12 hour, 18^o^C days with supplemental greenhouse lighting. To avoid cross-contamination, plants were spaced >12 cm apart and watered twice daily as needed with carbon-block filtered water provided via ultra-fine mist irrigation. Pots containing seedlings that failed to germinate and establish were replaced with a healthy germinated seedling of the same host genotype from the extra stock planted alongside the primary cones.

Once the first true leaf was fully extended (one month, post planting), plants received 900 uL of either rhizobium inoculum or a sterile water control. Each *Mesorhizobium* strain was cultured in two aliquots of 1 mL of TY broth in a 2 mL Eppendorf tube for 72 hours at 30^o^C at 300 rpm, with tubes oriented horizontally on the shaker. Immediately before inoculation, rhizobium cultures were centrifuged and resuspended to 10^7^ cells/mL in water (based on OD_600_). Starting seven days post inoculation, 1 mL of 1X strength modified low-nitrogen Fahraeus solution containing 7 ppm nitrogen as ammonium nitrate, was applied once a week to each pot. Post-inoculation, plants grew for one month, then shoots were clipped and dried to constant mass at 60^o^C and weighed. Belowground tissue was washed, frozen, and subsequently thawed and the nodules on each root system were counted. 1-2 mg of apical bud leaf tissue was submitted to an external laboratory for nitrogen composition on an elemental analyzer.

*Analysis*. To determine whether closely related strains exhibited genetic variation for benefits conferred to the host plant and the number of nodules formed on the host plant, we used a mixed effects general linear modelling approach (lme4, (3)). Rhizobium strain and plant genotype were included as random effects, and soil type, reserve, and block were included as fixed effects. Because of low replication for genotype interactions, we did not assess interactive effects. Assumptions of normality and homogeneity of variance in this and all subsequent linear models were assessed graphically (4). The significance of both random effects was determined with the likelihood ratio statistic calculated as the difference in -2ln between models differing in the inclusion versus exclusion of each random effect. This statistic was compared to a chi-squared distribution with one degree of freedom, which is generally conservative for random effects (5). Significance of each fixed effect was assessed with an F-test using type III sums of squares (lmerTest, (3)). The proportion of total variance explained by each random effect in a model was calculated in analogous models fit by restricted maximum likelihood. We used PCR to check each strain for expected presence/absence patterns for a portion of the *nodA* symbiosis gene (see below).

***II. Genomic attributes predicting cooperation & pan-genomics of the symbiosis island***

*Symbiosis genes.* We identified high-confidence symbiosis genes in the draft genomes of 48 wild *Mesorhizobium* strains from BioProject PRJNA171524 using orthology-based annotations from (1). This set includes 38 strains from a focal recombining population as well as 10 diverse strains that are distantly related to the focal population (1). We defined high-confidence symbiosis genes as those genes that 1) impact symbiosis and nitrogen fixation based on comparative study of *Mesorhizobium* (104 genes; (6)), or impact horizontal gene transfer of the symbiosis island (SI) (44 genes with 41 unique accessions; (7)), and 2) are among the 601 protein coding genes that map within the physical boundaries of the SI in the completed reference genome of *Mesorhizobium loti* strain MAFF303099 (8). The final list consists of MAFF303099 symbiosis island genes that are annotated and well documented in scientific literature as being either symbiosis-related or HGT-related. This list is conservative rather than exhaustive, and was used to determine presence/absence of the SI within our focal population.

*Genomic attributes predicting cooperation.* To examine whether the number of symbiosis genes present in a draft genome or the total size of a draft genome predicts the level of cooperation exhibited by the 38 closely related wild *Mesorhizobium* strains (above), and to test whether the number of symbiosis genes predicts the total size of a draft genome, we used three one-way ANOVAs (lm, (9)).

*Comparative Genomic Analysis.* The bimodal distribution of the number of predicted SI genes within a strain delineates two categories of genomes within the focal clade, and three categories total: 1) 32 focal strains with the SI (*focal_SI+*), 2) 6 focal strains without the SI (*focal_*SI-), 3) and 10 nonfocal strains with the SI (*nonfocal_*SI+). To identify genomic regions with a profile consistent with recent HGT we conducted LASTZ genome alignments (Fig 1), which report the average sequence identity within consecutive alignment blocks between in/dels, and identifies sequences flanking concatenated contig gaps as separate blocks (10).

To run a single LASTZ comparison per genome, prior to alignments all contigs from each *focal_SI+* strain assembly were concatenated to form a single pseudomolecule. To identify the best alignment between each *focal_SI+* strain and each *non-focal_SI+* strain throughout the genome, a custom pipeline reported the highest block sequence similarity shared at every base pair in the genome (https://github.com/jfaberha/lastz_lav_expansion). These highly conserved sequence blocks were then conservatively masked (ie removed) if they were found in alignments between genomes of any *focal_SI+* and any *focal_SI-* strain, in order to identify genomic intervals highly conserved between distantly related strains but with no homolog in related strains lacking the SI. We extracted conserved sequence blocks from LASTZ alignment output files using a custom pipeline that reported the highest sequence similarity for every base pair in pairwise genome comparisons (https://github.com/jfaberha/lastz_lav_expansion). Using a series of custom filters and masking parameters, we extracted the genomic regions from each *focal_SI+* strain that shares at least 95% sequence identity with *nonfocal_SI+* strains and shares no alignment of ≥85% sequence identity with any *focal_SI-* strain (https://github.com/jfaberha/lastz_lav_expansion). These thresholds were set based on empirical observations showing that *focal_SI+* and *nonfocal_SI+* strains are distantly related genome-wide (~87.5% ANI for core genomic genes) but show strong sequence conservation in putative SI genes (>95% ANI), consistent with recent horizontal gene transfer (HGT), and *focal-island* and *focal_SI-* strains are from the same recombining *Mesorhizobium* population (~97.5% ANI across their core genome; (1)) although SI regions appear as deletions in *focal_SI-* strains/insertions in *focal_SI+* strains. The conservative ≥85% masking parameter may have excluded some genes without true homologs in the *focal_SI-* strains but gave us high confidence that identified genes are exclusive to SI+ strains.

These candidate SI intervals were converted to bed files and BedTools v2.24.0 (11) was used to extract concatenated *focal_SI+* strain genome assemblies in fasta format. Using Bowtie2 v.2.2.6 (12), we aligned all annotated genes from each respective genome to the extracted genomic intervals, and all perfect alignments were considered HGT and/or SI genes. We compiled a list of all genes found in HGT regions of *focal_SI+* strains and looked at presence/absence data for that list as defined in (1). The presence/absence table included information for all strains from each category in addition to 6 *Mesorhizobium* reference/outgroup species downloaded from NCBI (1). Candidate HGT genes were clustered based on presence/absence patterns across all genomes using a heatmap dendrogram (gplots: heatmap2 (13)) with Ward.D2 clustering and Euclidean distance methods (14). This allowed us to distinguish clusters of HGT genes with highly variable presence/absence patterns from those likely coinherited and present in nearly all SI+ strains, which we define as “near-core” SI genes.

To examine the presence/absence profile of particular SI-related gene categories in the full set of strains, we constructed two analogous heatmaps with high confidence symbiosis gene lists from (6) and (7). While there is overlap between each of these lists and the candidate HGT gene list, these lists included some genes present in *focal_SI-* strains. We generated bootstrap values for dendrogram gene clusters using 1000 clustering iterations in pvclust (15).

To nominate functional categories of genes enriched in the putative SI and other recent HGT genes, we ran gene ontology (GO) enrichment analysis on full HGT gene set and the near-core SI genes. These sets of genes were compared to remaining genes in the full annotated *Mesorhizobium* pangenome in Blast2GO v3.2 (16) with an FDR cutoff of 0.05. Here, the pangenome is defined as all genes annotated in the reference *M. loti* strain MAFF303099 plus additional *de novo* genes annotated in focal range *Mesorhizobium* strains.

*Impact of SI absence on plant fitness.* To examine whether the presence or absence of the SI predicts plant biomass and thus a strain’s level of cooperation, we used a one-way ANOVA (lm, (9)). While six SI- strains were identified bioinformatically, the stock culture of one of these strains was contaminated, so we tested whether the remaining five SI- strains have impacts on host fitness that is indistinguishable from the no-rhizobium inoculum control treatment, using a fixed effects general linear model (lm, (9)) on a subset of the data including only these five strains and the negative control plants. We included three fixed effects terms in the model: inoculum (6 levels: the no-rhizobium control treatment, and each of the five strains lacking the SI for which we had cooperation data), host genotype (3 levels), and block (2 levels), and tested for their effects on shoot mass. We log-transformed shoot mass to better meet the assumption of homogeneity of variance. The no-rhizobium control treatment served as the reference level for the inoculum factor, so significance values for each SI- strain reflects whether it yields a shoot value different from that of the no-rhizobium control. Significance values were corrected for 5 multiple tests using Holms’ sequential Bonferroni procedure (17).

***III. Patterns of relatedness for the SI***

To determine patterns of relatedness among strains for regions we identify as horizontally transferred, we calculated average nucleotide identity (*ANI*) for all genes within and between different groups of strains. First, we generated multiple sequence alignments (MSA) in fasta format and distance matrices for each core genomic gene (single-copy genes found in 100% of strains) and each of the 177 near-core SI genes for all strains using ClustalO v1.1.0 (18). Using the ClustalO distance matrices, we calculated *ANI* separately for genomic and SI genes both among the focal clade strains and between the focal clade (*focal-island/focal-non-island*) and *non-focal-island* strains for a total of four *ANI* summary values. The distances from these tables were converted to sequence similarity values using the formula *ANI = (1 - D) x 100*, with D being the distances in the matrix. Each *ANI* summary value was weighted by the length of a gene’s MSA.

To identify patterns of coinheritance of genes, we clustered genes based on pairwise sequence similarities for each set of orthologs among strains (gplots heatmap2, (13)). For each near-core gene, we calculated sequence similarity based on pairwise comparisons of all orthologs from SI*+* strains for a total of 861 comparisons (n=42 SI+ strains for n*[n-1]/2=861). Sequence similarity values for all near-core genes were compiled into a matrix and clustered using the Ward.D2 clustering and Euclidean distance methods (14). Bootstrap values were generated with 1000 clustering iterations (pvclust, (15)), and gene clusters with bootstrap values ≥90 were inferred to be statistically significant.

To verify the accuracy of SI gene clustering results, we used RAxML v8.2.10 (19) to create unrooted maximum likelihood trees for each near-core HGT gene and used TOPD v4.6 (20) to run pairwise tree topology comparisons for all 177 genes. The model used for RAxML was GTRGAMMA, and the best trees for each gene were determined from 100 algorithm iterations. We compared the overall tree topologis and the distance between nodes among each tree by calculating split distance in TOPD (21), an index based on the number of shared partitions between trees, and nodal distance, a metric of path-length between node placement on each tree (21, 22). Next, we summarized split distance and nodal distance for topology comparisons for genes within and between significant gene clusters identified by pvclust. To do so, we ranked split distances and nodal distances for all tree comparisons from more similar to less similar, then ran non-parametric one-way Mann–Whitney *U* tests to check whether our within-cluster tree comparisons are statistically different from between-cluster comparisons, with our prediction being within-cluster tree comparisons would show more topological similarity.

We also aimed to compare the phylogenetic profiles of genomic and SI genes and examine variation in tree topologies among SI genes. Using MSAs generated by ClustalO, we built neighbor networks for a concatenated set of 100 random core-genome genes and concatenated clusters of near-core HGT genes (identified with pvclust from pairwise percent identity matrices) using SplitsTree (23). MSA concatenation was performed with the program FASconCATv1.0.pl (24). Strains missing a particular gene were included in the respective MSA with “-” characters representing gaps in place of aligned sequence. With a series of custom scripts, we removed strains from each concatenated MSA with more than 75% of the sequence length consisting of “-” characters.

***IV. Stability of the SI under laboratory conditions***

To assess whether the SI within *Mesorhizobium* strains is stable or subject to loss under the culture conditions used in the laboratory, we selected at random eight SI+ strains (NJ1, SH9, SJ6, SH7, SM3, SJ2, SM2, SJ7), and four SI- strains (NJ5, NJ3, NM5, SH8) as negative controls to undergo successive rounds of culturing. To mimic conditions under which strains were isolated and cultured prior to draft genome sequencing (1, 25), three replicates of each strain were cultured in 1 ml tryptone-yeast broth (26) inoculated from cryopresered stocks, and incubated at 30 °C with 300 rpm. Once a week for four weeks (~45-170 generations (27)) cell density was estimated from optical density at A_600_, and fresh cultures were seeded with approximately 10^3^ cells from the previous culture. Each week, 10 μL of evolved culture of each replicate was diluted to a range between 1:10^3^ and 1:10^6^, in a 96-well plate with 90 μL TYB. Then 100 μL of diluted culture was spread on tryptone yeast agar plates (26) using sterile glass beads. Plates were incubated at 30 °C until visible colonies formed, and cells were collected from individual colonies to use in a PCR assay consisting of the robust primer set, *nodA* (nodA_69F: CGC CGA GTT CTT TCG TGA TA; nodA_390R: TCC GAA CCT CTC AAC ATG ATT C). The symbiosis gene *nodA* is conserved in all SI+ strains and partial *nodA* sequence was the target of colony PCR for 25 colonies from replicate 1 of each SI+ strain. Primers amplifying partial 16S rRNA sequence (25) from the main chromosome were used as a methodological control. In colony PCR, cells from single colonies were suspended in 10 μL 0.5x TYB and 1 μL served as template DNA in the 10 μL PCR. This cycled in a thermocycler with parameters set to 3 min at 95 °C, followed by 35 cycles of 20 s at 92 °C for denaturation, 20 s at 56 °C for annealing, and 2 min at 68 °C for extension, then an additional 3 min at 68 °C. PCR products were visualized under UV light, on a 1.2% agarose gel pre-stained with SafeView Classic.

To verify that the isolates after 4 weeks of continuous culture were uncontaminated, PCR products from two tested colonies from each of the eight SI+ and two SI- strains were Sanger sequenced. The sequences were aligned and trimmed in Jalview (28) and compared with the original isolate’s *nodA* and/or *16S* sequence.

**REFERENCES**

1. Porter SS, Chang PL, Conow CA, Dunham JP, Friesen ML. Association mapping reveals novel serpentine adaptation gene clusters in a population of symbiotic Mesorhizobium. Isme J [Internet]. 2017; Available from: http://eutils.ncbi.nlm.nih.gov/entrez/eutils/elink.fcgi?dbfrom=pubmed&id=27420027&retmode=ref&cmd=prlinks

2. Porter SS. Adaptive divergence in seed color camouflage in contrasting soil environments. New Phytol. 2013 Mar 1;197(4):1311–20.

3. Bates D, Mächler M, Bolker B, Walker S. Fitting Linear Mixed-Effects Models Using lme4. J Stat Soft. 2015;67(1):1–48.

4. Zuur A. Mixed Effects Models and Extensions in Ecology with R [Internet]. New York, New York, USA: Springer-Verlag; 2009 [cited 2018 Jan 23]. Available from: //www.springer.com/us/book/9780387874579

5. Pinheiro JC, Bates D. Mixed-Effects Models in S and S-PLUS. Springer Science & Business Media; 2009. 538 p.

6. Laranjo M, Alexandre A, Oliveira S. Legume growth-promoting rhizobia: An overview on the Mesorhizobium genus. Microbiological Research. 2014;169(1):2–17.

7. Haskett TL, Terpolilli JJ, Bekuma A, O’Hara GW, Sullivan JT, Wang P, et al. Assembly and transfer of tripartite integrative and conjugative genetic elements. Proceedings of the National Academy of Sciences. 2016;113(43):12268–73.

8. Uchiumi T, Ohwada T, Itakura M, Mitsui H, Nukui N, Dawadi P, et al. Expression Islands Clustered on the Symbiosis Island of the Mesorhizobium loti Genome. J Bacteriol. 2004 Apr 15;186(8):2439–48.

9. Team RC. R: A Language and Environment for Statistical Computing. 2015; Available from: https://www.R-project.org/

10. Harris, Robert. Improved pairwise alignment of genomic DNA, PhD Dissertation [Internet]. Pennsylvania State University; 2007 [cited 2018 Mar 15]. Available from: https://search.proquest.com/openview/bc77cca0fb9390b44b9ef572fb574322/1?pq-origsite=gscholar&cbl=18750&diss=y

11. Quinlan AR, Hall IM. BEDTools: a flexible suite of utilities for comparing genomic features. Bioinformatics. 2010 Mar 15;26(6):841–2.

12. Langmead B, Salzberg SL. Fast gapped-read alignment with Bowtie 2. Nature Methods. 2012;9(4):357–359.

13. Warnes GR, Bolker B, Bonebakker L, Gentleman R, Huber W, Liaw A, et al. gplots: Various R programming tools for plotting data. R package version. 2009;2(4):1.

14. Ward JH. Hierarchical Grouping to Optimize an Objective Function. Journal of the American Statistical Association. 1963 Mar 1;58(301):236–44.

15. Suzuki R, Shimodaira H. Pvclust: an R package for assessing the uncertainty in hierarchical clustering. Bioinformatics. 2006 Jun 15;22(12):1540–2.

16. Conesa A, Gotz S, Garcia-Gomez JM, Terol J, Talon M, Robles M. Blast2GO: a universal tool for annotation, visualization and analysis in functional genomics research. Bioinformatics. 2005;21(18):3674–6.

17. Holm S. A Simple Sequentially Rejective Multiple Test Procedure. Scandinavian Journal of Statistics. 1979;6(2):65–70.

18. Sievers F, Wilm A, Dineen D, Gibson TJ, Karplus K, Li W, et al. Fast, scalable generation of high‐quality protein multiple sequence alignments using Clustal Omega. Molecular Systems Biology. 2011 Jan 1;7(1):539.

19. Stamatakis A. RAxML version 8: a tool for phylogenetic analysis and post-analysis of large phylogenies. Bioinformatics. 2014;30(9):1312–1313.

20. Puigbò P, Garcia-Vallvé S, McInerney JO. TOPD/FMTS: a new software to compare phylogenetic trees. Bioinformatics. 2007 Jun 15;23(12):1556–8.

21. Robinson DF, Foulds LR. Comparison of phylogenetic trees. Mathematical Biosciences. 1981 Feb 1;53(1):131–47.

22. Steel MA, Penny D. Distributions of Tree Comparison Metrics-Some New Results. Systematic Biology. 1993;42(2):126–41.

23. Huson DH. Application of phylogenetic networks in evolutionary studies. Mol Biol Evol. 2006;23(2):254–67.

24. Kück P, Meusemann K. FASconCAT: Convenient handling of data matrices. Molecular Phylogenetics and Evolution. 2010;56(3):1115–1118.

25. Porter SS, Rice KJ. Trade-offs, spatial heterogeneity, and the maintenance of microbial diversity. Evolution. 2013;67(2):599–608.

26. Somasegaran P, Hoben HJ. Handbook for Rhizobia - Methods in Legume-Rhizobium | Padma Somasegaran | Springer [Internet]. New York, New York, USA: Springer-Verlag; 1994 [cited 2018 Jan 24]. Available from: http://www.springer.com/us/book/9781461383772

27. Chen Wen Xin, Wang En Tao, David Kuykendall L. Mesorhizobium. Bergey’s Manual of Systematics of Archaea and Bacteria [Internet]. 2015 Apr 17 [cited 2018 Mar 22]; Available from: https://onlinelibrary.wiley.com/doi/abs/10.1002/9781118960608.gbm00839

28. Waterhouse AM, Procter JB, Martin DMA, Clamp M, Barton GJ. Jalview Version 2--a multiple sequence alignment editor and analysis workbench. Bioinformatics. 2009 May 1;25(9):1189–91.

FIGURES

**Figure 1. Strategy for detection of symbiosis island using LASTZ.** Conceptual diagram illustrating the bioinformatic approach by which LASTZ alignments were used to identify the symbiosis island from sequential pair-wise alignments of contigs among draft genomes. *Alignment of M. loti R7A SI sequence serves as independent verification of pipeline and does not directly inform detection of the SI in the focal population.
