# Supplementary material for: Dynamic genomic architecture of mutualistic cooperation in a wild population of Mesorhizobium
Source: ISME J. 2018 Sep 14;13(2):301–15. doi: 10.1038/s41396-018-0266-y (PMC6331556; doi:10.1038/s41396-018-0266-y)
Supplement: Supplementary file 2 — Supplementary Information 2 [file 41396_2018_266_MOESM2_ESM.docx]

**Supplementary Information 2**

**RESULTS**

PCR and sequence data indicate that the symbiosis island is retained in our wild *Mesorhizobium* strains despite passage through serial cultures in the laboratory. Electrophoresis of PCR products show none of the eight SI positive strains of *Mesorhizobium* have ejected the SI after four weeks of serial transfer in Eppendorf cultures (~45-170 generations). Sanger sequencing of a subsample of these PCR products confirmed them to be the target *nodA* and *16S* sequences.

**TABLES**

| **Table 1:** PCR results for eight SI+ and two SI- strains after 4 weeks of laboratory culturing | | | | | | |
| --- | --- | --- | --- | --- | --- | --- |
| **Isolate** | **Colonies tested** | ***nod A* results** | ***16S* results** |  |  |  |
| NJ1 | 25 | 25 SI+ | 16S + |  |  |  |
| SH9 | 25 | 25 SI+ | 16S + |  |  |  |
| SJ6 | 25 | 25 SI+ | 16S + |  |  |  |
| SH7 | 25 | 25 SI+ | 16S + |  |  |  |
| SM3 | 25 | 25 SI+ | 16S + |  |  |  |
| SJ2 | 25 | 25 SI+ | 16S + |  |  |  |
| SM2 | 25 | 25 SI+ | 16S + |  |  |  |
| NJ7 | 25 | 25 SI+ | 16S + |  |  |  |
| NJ5 | 4 | 4 SI- | 16S + |  |  |  |
| NJ3 | 4 | 4 SI- | 16S + |  |  |  |

| **Table 2.** Sanger sequencing of partial *16S* loci for two of the 25 colonies amplified in PCR. Each of the 8 strains that contained the symbiosis island after 4 weeks of repeated subculturing were compared with the original isolate sequence. Isolate, the original strain containing the symbiosis island; Colony, the randomly selected colony from those used in the PCR assay for which sanger sequence was generated; SNPs, the number of single nucleotide differences between the sequence for the ancestral strain and the descendent after 4 weeks; Comparison, the number of base pairs (Bp) within the *16S* gene that were compared. | | | | |
| --- | --- | --- | --- | --- |
| **Isolate** | **Colony** | **SNPs** | **Comparison (Bp)** |  |
| NJ1 | NJ1.1 | 2 | 513 |  |
| NJ1 | NJ1.15 | 0 | 513 |  |
| SH9 | SH9.5 | 1 | 529 |  |
| SH9 | SH9.9 | 1 | 529 |  |
| SJ6 | SJ6.1 | 0 | 705 |  |
| SJ6 | SJ6.4 | 0 | 705 |  |
| SH7 | SH7.1 | 0 | 541 |  |
| SH7 | SH7.2 | 0 | 541 |  |
| SM3 | SM3.3 | 1 | 574 |  |
| SM3 | SM3.5 | 1 | 574 |  |
| SJ2 | SJ2.4 | 1 | 640 |  |
| SJ2 | SJ2.6 | 0 | 640 |  |
| SM2 | SM2.1 | 1 | 608 |  |
| SM2 | SM2.8 | 0 | 608 |  |
| NJ7 | NJ7.7 | 0 | 616 |  |
| NJ7 | NJ7.9 | 0 | 616 |  |
| NJ5 | NJ5.1 | 1 | 560 |  |
| NJ5 | NJ5.2 | 0 | 560 |  |
| NJ3 | NJ3.1 | 0 | 643 |  |
| NJ3 | NJ3.4 | 0 | 643 |  |

| **Table 3:** Sanger sequencing of partial *nodA* loci for two of the 25 colonies amplified in PCR. Each of the 8 strains that contained the symbiosis island after 4 weeks of repeated subculturing were compared with the original isolate sequence. Isolate, the original strain containing the symbiosis island; Colony, the randomly selected colony from those used in the PCR assay for which sanger sequence was generated; SNPs, the number of single nucleotide differences between the sequence for the ancestral strain and the descendent after 4 weeks; Comparison, the number of base pairs (Bp) within the nodA gene that were compared. | | | | |
| --- | --- | --- | --- | --- |
| **Isolate** | **Colony** | **SNPs** | **Comparison** |  |
| NJ1 | NJ1.1 | 0 | 281 |  |
| NJ1 | NJ1.15 | 0 | 281 |  |
| SH9 | SH9.5 | 0 | 293 |  |
| SH9 | SH9.9 | 0 | 293 |  |
| SJ6 | SJ6.1 | 0 | 283 |  |
| SJ6 | SJ6.4 | 0 | 283 |  |
| SH7 | SH7.1 | 0 | 277 |  |
| SH7 | SH7.2 | 1 | 277 |  |
| SM3 | SM3.3 | 0 | 273 |  |
| SM3 | SM3.5 | 0 | 273 |  |
| SJ2 | SJ2.4 | 2 | 253 |  |
| SJ2 | SJ2.6 | 0 | 253 |  |
| SM2 | SM2.1 | 0 | 290 |  |
| SM2 | SM2.8 | 0 | 290 |  |
| NJ7 | NJ7.7 | 0 | 277 |  |
| NJ7 | NJ7.9 | 0 | 277 |  |

**FIGURES**


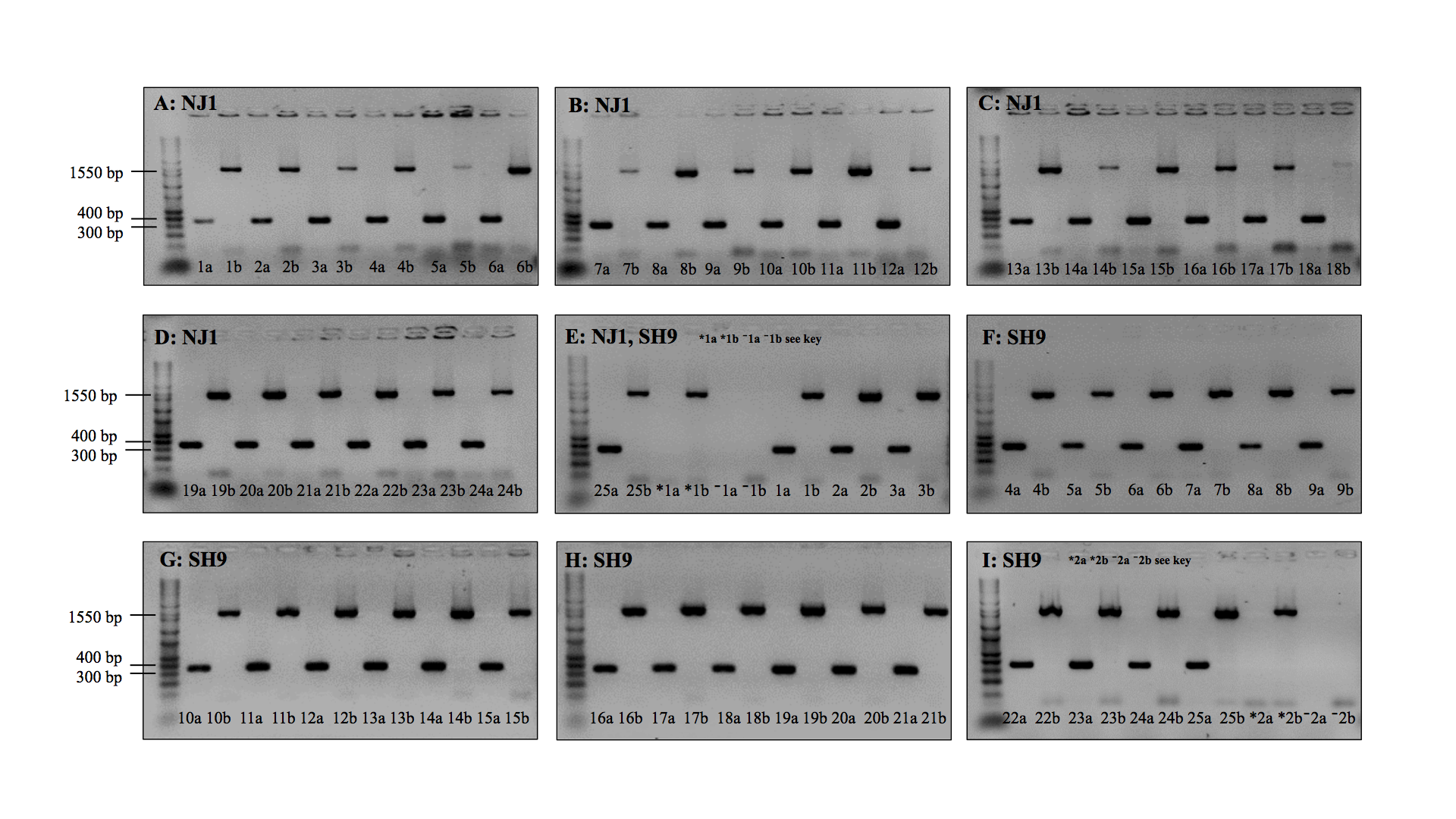

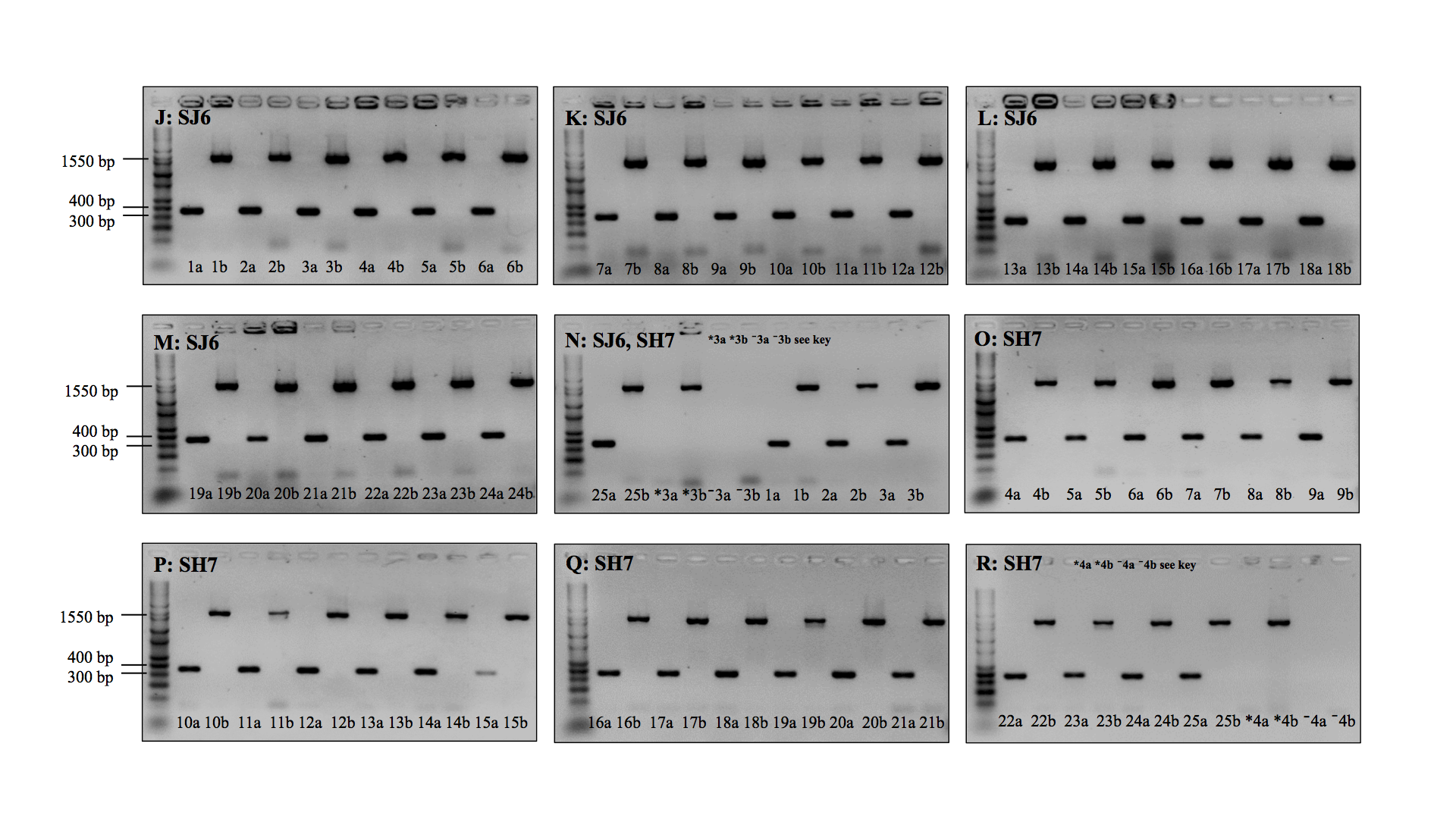


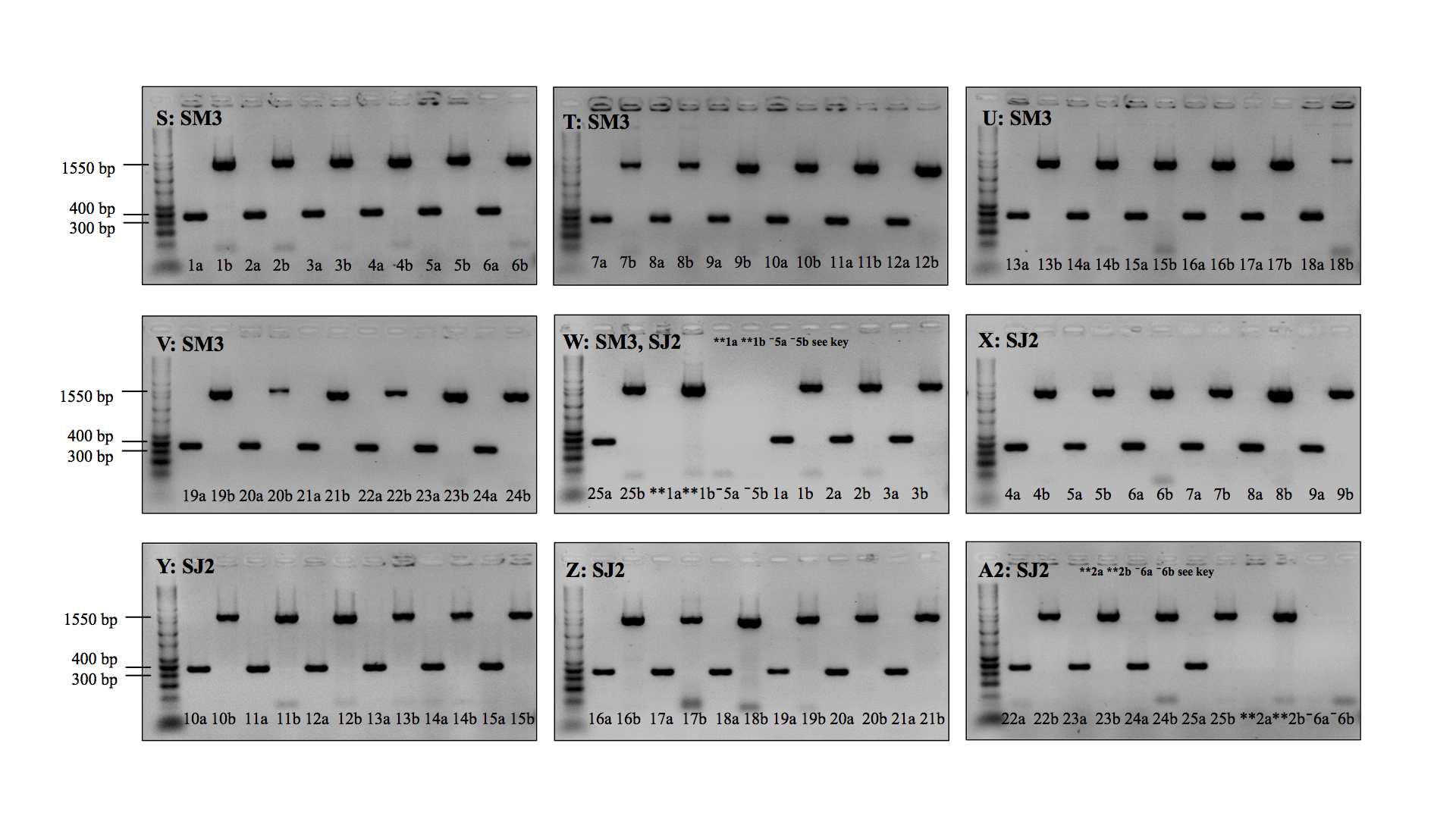

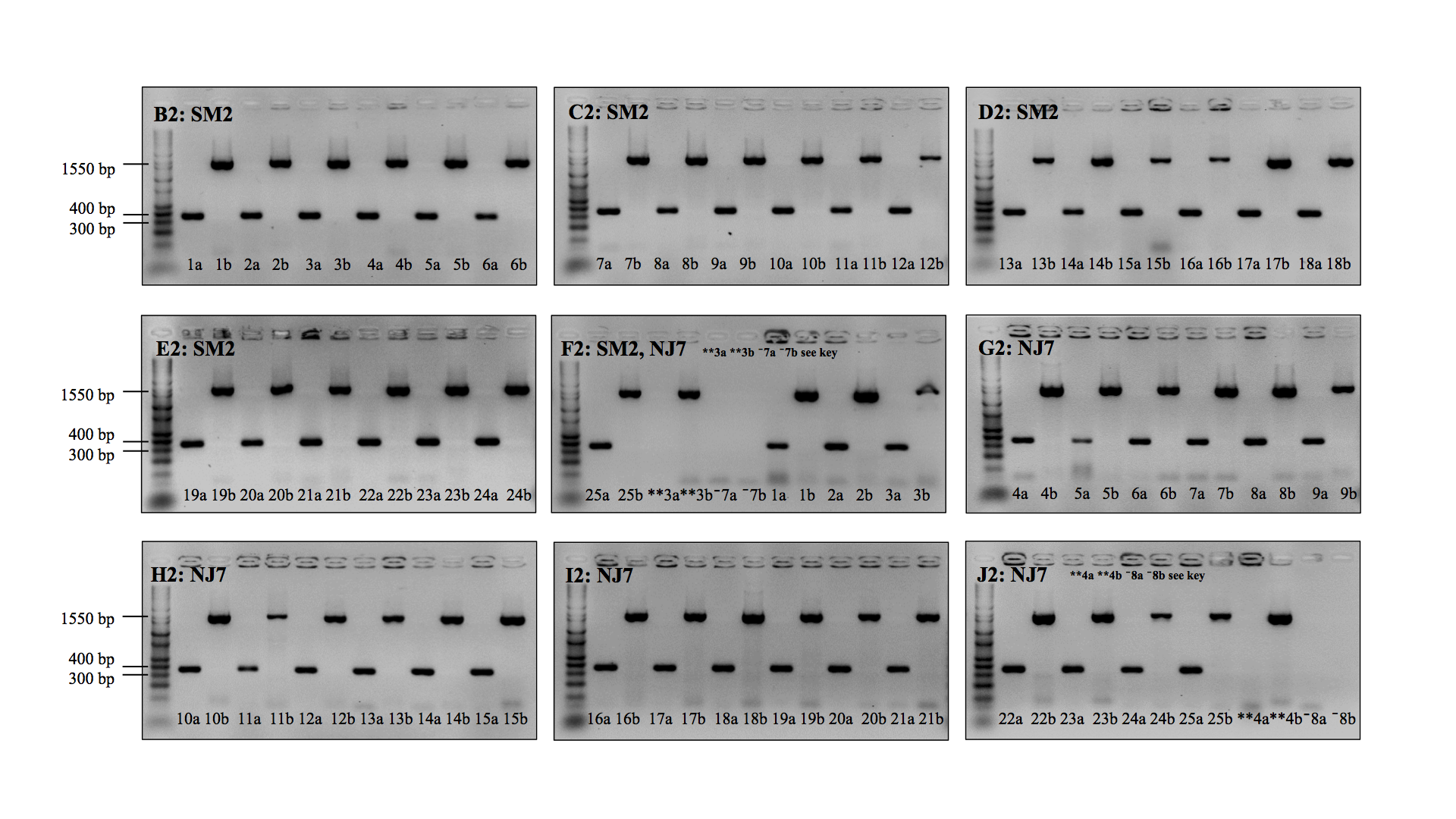


**Figure 1. Electrophoresis Images of PCR Products.** Panels A-J2 show eight ancestral SI+ strains and two SI- strains screened for the symbiosis island in a PCR assay, after 4 weeks of continuous culture via serial transfer in the laboratory. Twenty-five colonies of eight ancestral SI+ strains (NJ1, SH9, SJ6, SH7, SM3, SJ2, SM2, SJ7), and four colonies of two ancestral SI- strains (NJ5 *1 - *4 and NJ3 **1 - **4) (Negative (water) controls ̄ 1 - ̄ 8), were tested using the *NodA* (a) primer set for the symbiosis island, and *16S* (b) primer set for the 16s ribosomal subunit on the main *Mesorhizobium* chromosome.
