# Supplementary material for: Dynamic genomic architecture of mutualistic cooperation in a wild population of Mesorhizobium
Source: ISME J. 2018 Sep 14;13(2):301–15. doi: 10.1038/s41396-018-0266-y (PMC6331556; doi:10.1038/s41396-018-0266-y)
Supplement: Supplementary file 3 — Supplementary Information 3 [file 41396_2018_266_MOESM3_ESM.docx]

**Supplementary Information 3.** Results of multiple comparisons of means (*a priori* contrasts) for shoot mass conferred by each of five SI- strains (NJ9, NJ3, NM5, SH8, NJ5) vs. water-only inoculated control plants (neg).

Once contrasts between these means (lme4, [1]); are corrected for five multiple tests using via Holm’s Sequential Bonferroni procedure (multcomp, [2]), the impact of individual SI- strains on shoot mass are indistinguishable from that of the uninoculated plants. We note that low numbers of replicates per strain make such comparisons for individual strains low-powered tests.

Simultaneous Tests for General Linear Hypotheses

Multiple Comparisons of Means: User-defined Contrasts

Fit: lmer(formula = log(Shoot Mass) ~ Rhizobial treatment + (1 | Host genotype) + Block)

**Linear Hypotheses Estimate Std. Error z value Pr(>|z|)**

NJ9 - neg == 0 0.15689 0.12200 1.286 0.794

NJ3 - neg == 0 -0.13125 0.12200 -1.076 0.846

NM5 - neg == 0 -0.10273 0.12200 -0.842 0.846

SH8 - neg == 0 -0.25248 0.12200 -2.070 0.192

NJ5 - neg == 0 -0.07435 0.12200 -0.609 0.846

(Adjusted p values reported -- holm method)

**References**

1. Bates D, Mächler M, Bolker B, Walker S. Fitting Linear Mixed-Effects Models Using lme4. *J Stat Softw* 2015; **67**: 1–48.

2. Hothorn T, Bretz F, Westfall P. Simultaneous Inference in General Parametric Models. *Biom J* 2008; **50**: 346–363.
