# Supplementary material for: Dynamic genomic architecture of mutualistic cooperation in a wild population of Mesorhizobium
Source: ISME J. 2018 Sep 14;13(2):301–15. doi: 10.1038/s41396-018-0266-y (PMC6331556; doi:10.1038/s41396-018-0266-y)
Supplement: Supplementary file 4 — Supplementary Information Legend [file 41396_2018_266_MOESM4_ESM.docx]

**SUPPLEMENTARY INFORMATION LEGEND**

**Supplementary Information 1.** Detailed version of the methods.

**Supplementary Information 2.** Evidence that the symbiosis island is retained despite passage through serial cultures in the laboratory.

**Supplementary Information 3.** Results of multiple comparisons of means between the five SI- strains for which we estimated impacts on host shoot mass as compared to uninoculated plants.

**Table S1. Strains used in the study.**

**Table S2. Plant genotypes used in the study.**

**Table S3.** **Horizontally transferred genes in SI+ strains.** For all genes detected in the HGT pipeline, table lists summary information and statistics, annotations, and loci within respective genome assemblies of each strain.

**Table S4.** **GO enrichment results for HGT genes, symbiosis island genes, and individual genes clusters.** GO enrichment analysis was performed for 177 near-core SI genes, the remaining 1186 rare HGT genes, and near-core genes clusters with a minimum of 10 genes using Blast2GO.

**Table S5. Symbiosis genes that fall outside of the detected symbiosis island.** Genes with gene ontology annotation “symbiosis, encompassing mutualism through parasitism” (GO:0044403) found in strains from the focal strain that lack the symbiosis island and their frequencies (%) among groups of strains in our study.

**Table S6. Genes associated with cooperation.**Accessory genes within the *focal SI+* population whose presence/absence correlated with cooperation phenotype; note that none of these genes fell within our SI gene list.

**Table S7.** **Presence/absence patterns for putative HGT genes in SI+ strains.** For all genes detected in HGT pipeline, table lists summary information and statistics, annotations, and presence/absence for each.

**Figure S1.** **Presence/absence profiles of the 1363 putative horizontally transferred (HGT) genes.** Ward.D2 clustering shows a major bifurcation between rare HGT genes and *near-core HGT genes. Red bars indicate genes present in the symbiosis island of the *Mesorhizobium loti* MAFF303099 reference genome and are high confidence symbiosis island genes in *Mesorhizobium*. **Focal strains lacking the symbiosis island (*focal*_SI-).

**Figure S2.** **Heatmap showing pairwise sequence identity among near-core symbiosis island genes.** While these genes are hypothesized to be part of symbiosis island in focal geographic range, different clusters of genes show variable patterns of relatedness among SI+ strains, which indicates contrasting evolutionary histories for different sets of genes. The x-axis represents all pairwise comparisons for orthologs between 42 SI+ strains (861 comparisons). The y-axis represents all 177 near core SI genes. Ward clustering groups near-core SI genes with similar profiles of pairwise ortholog sequence identity among strains, with color-coded bars to the right of the heat map showing significant clusters of genes supported by bootstrap values ≥90. Asterisk; Cluster 10 contains *vir* genes, which are implicated in symbiosis island HGT but not found in the *Mesorhizobium loti* MAFF303099 symbiosis island.
