# Supplementary figures and images for: Dynamic genomic architecture of mutualistic cooperation in a wild population of Mesorhizobium
Source: ISME J. 2018 Sep 14;13(2):301–15. doi: 10.1038/s41396-018-0266-y (PMC6331556; doi:10.1038/s41396-018-0266-y)

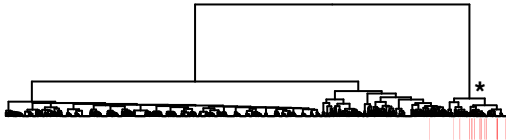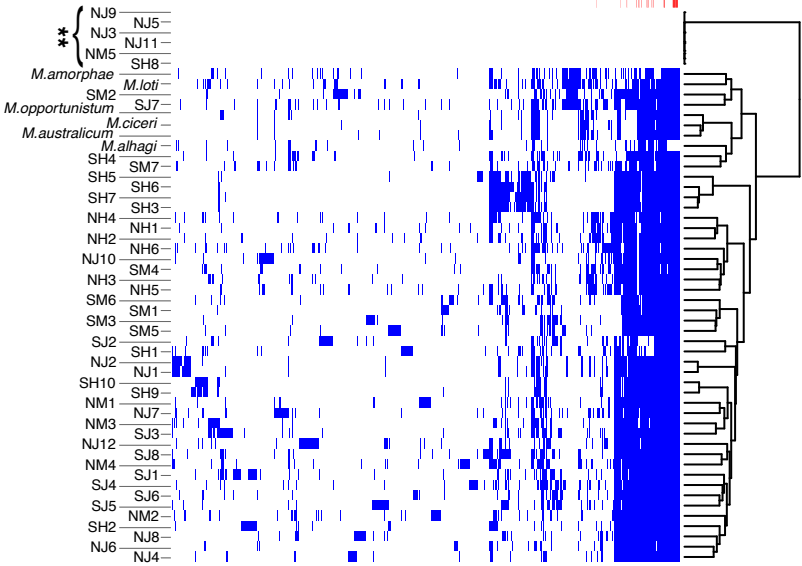

Candidate HGT Genes

Supplement: Supplementary file 5 — Figure S1 [file 41396_2018_266_MOESM5_ESM.pdf]
