# Supplementary material for: Dynamic genomic architecture of mutualistic cooperation in a wild population of Mesorhizobium
Source: ISME J. 2018 Sep 14;13(2):301–15. doi: 10.1038/s41396-018-0266-y (PMC6331556; doi:10.1038/s41396-018-0266-y)
Supplement: Supplementary file 11 — Table S5 [file 41396_2018_266_MOESM11_ESM.pdf]

All Strains(n=54)	Focal_SI+(n=32)	Nonfocal_SI+(n=	Focal_SI-(n=6)	ref spp.(n=6)	Seq. Accession	Seq. Description
**70.37**	**100**	**0**	**100**	**0**	**denovo000891**	**glycosyl transferase**
3.7	3.13	10	0	0	denovo003427	external scaffolding protein d
5.56	6.25	10	0	0	denovo003428	protein b
5.56	6.25	10	0	0	denovo003434	major spike protein g
5.56	6.25	10	0	0	denovo003435	major spike protein g
5.56	6.25	10	0	0	denovo003439	external scaffolding protein d
1.85	0	10	0	0	denovo007925	two-component response regulator
1.85	0	10	0	0	denovo007926	response regulator
3.7	0	20	0	0	denovo008733	glycosyl transferase
5.56	0	20	0	16.67	denovo009144	sulfate large subunit
7.41	0	20	0	33.33	denovo009145	sulfate large subunit
7.41	0	20	0	33.33	denovo009146	sulfate large subunit
1.85	0	10	0	0	denovo009629	two-component response regulator
1.85	3.13	0	0	0	denovo011176	feruloyl esterase
1.85	3.13	0	0	0	denovo012323	external scaffolding protein d
7.41	12.5	0	0	0	denovo012571	feruloyl esterase
1.85	0	10	0	0	denovo013321	transcriptional regulator protein
1.85	3.13	0	0	0	denovo013967	feruloyl esterase
22.22	0	100	0	33.33	GI:13472855	hypothetical protein mll3280
**100**	**100**	**100**	**100**	**100**	**GI:13474136**	**nodF**
**100**	**100**	**100**	**100**	**100**	**GI:13474137**	**nodE**
**79.63**	**71.88**	**100**	**66.67**	**100**	**GI:13474849**	**hypothetical protein msr8740**
3.7	0	10	0	16.67	GI:13475164	nolL
3.7	0	0	0	33.33	GI:13475296	nolX
5.56	0	0	0	50	GI:13475297	nolW
5.56	0	0	0	50	GI:13475299	nolT
5.56	0	0	0	50	GI:13475300	nolU
5.56	0	0	0	50	GI:13475301	nolV
**100**	**100**	**100**	**100**	**100**	**GI:13476217**	**hemolysin-like protein**
**100**	**100**	**100**	**100**	**100**	**GI:13476292**	**sulfate adenylyltransferase subunit 2**
**98.15**	**100**	**100**	**100**	**83.33**	**GI:13476293**	**adenylylsulfate kinase protein**
**100**	**100**	**100**	**100**	**100**	**GI:13476514**	**nodG**
1.85	0	0	0	16.67	GI:161621456	hypothetical protein mlr9393
