# Supplementary material for: Dynamic genomic architecture of mutualistic cooperation in a wild population of Mesorhizobium
Source: ISME J. 2018 Sep 14;13(2):301–15. doi: 10.1038/s41396-018-0266-y (PMC6331556; doi:10.1038/s41396-018-0266-y)
Supplement: Supplementary file 6 — Figure S2 [file 41396_2018_266_MOESM6_ESM.pdf]

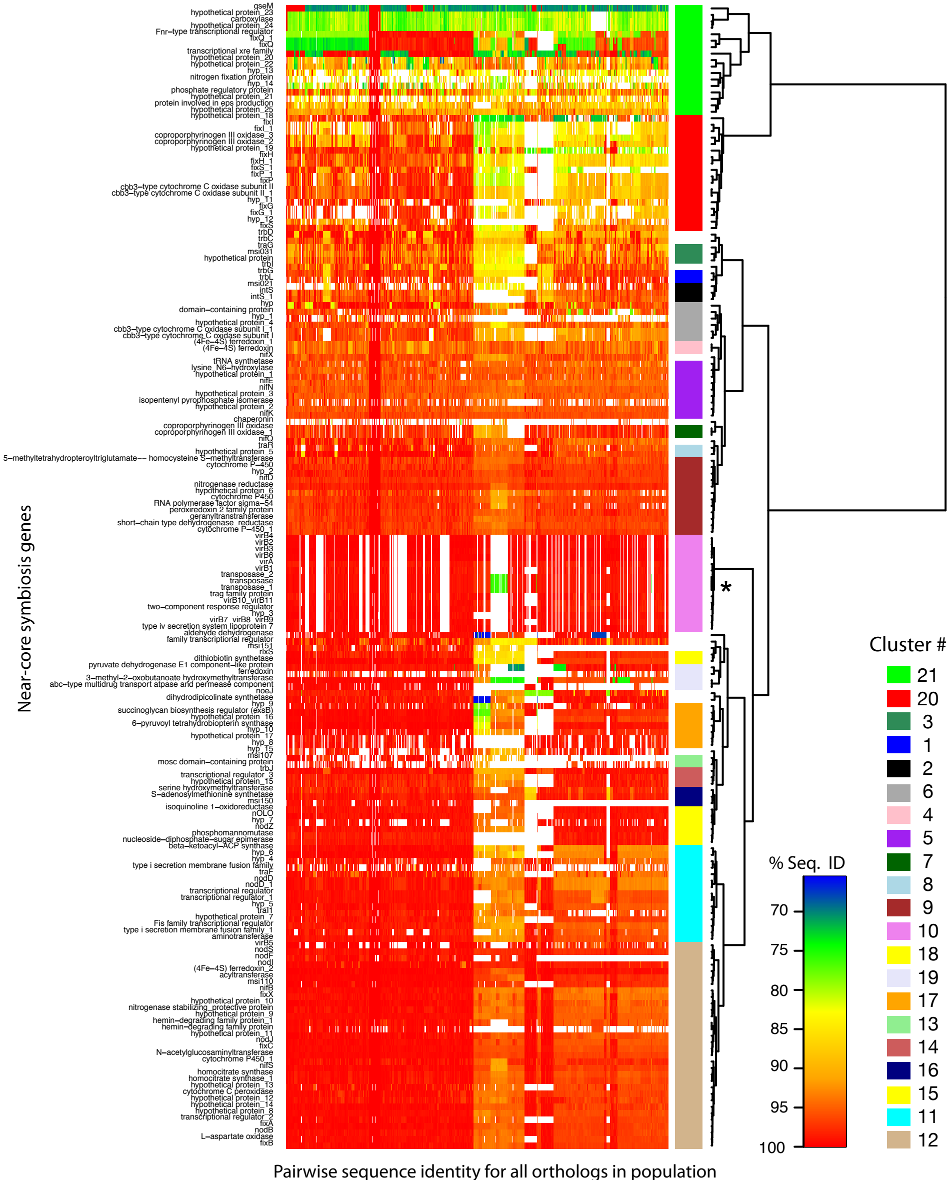
qseM
hypothetical protein_23
carboxylase
hypothetical protein_24
Fnr–type transcriptional regulator
fixQ_1
fixQ
transcriptional xre family
hypothetical protein_20
hypothetical protein_22
hyp_13
nitrogen fixation protein
hyp_14
phosphate regulatory protein
hypothetical protein_21
protein involved in eps production
hypothetical protein_25
hypothetical protein_18
fixI
fixI_1
coproporphyrinogen III oxidase_3
coproporphyrinogen III oxidase_2
hypothetical protein_19
fixH
fixH_1
fixS_1
fixP_1
fixP
cbb3–type cytochrome C oxidase subunit II
cbb3–type cytochrome C oxidase subunit II_1
hyp_11
fixG
fixG_1
hyp_12
fixS
trbD
trbC
traG
msi031
hypothetical protein
trbI
trbG
trbL
msi021
intS
intS_1
hyp
domain–containing protein
hyp_1
hypothetical protein_4
cbb3–type cytochrome C oxidase subunit I_1
cbb3–type cytochrome C oxidase subunit I
(4Fe–4S) ferredoxin_1
(4Fe–4S) ferredoxin
nifX
tRNA synthetase
lysine_N6–hydroxylase
hypothetical protein_1
nifE
nifN
hypothetical protein_3
isopentenyl pyrophosphate isomerase
hypothetical protein_2
nifK
chaperonin
coproporphyrinogen III oxidase
coproporphyrinogen III oxidase_1
nifQ
hypothetical protein_5
traR
5–methyltetrahydropteroyltriglutamate–– homocysteine S–methyltransferase
cytochrome P–450
hyp_2
nifD
nitrogenase reductase
hypothetical protein_6
cytochrome P450
RNA polymerase factor sigma–54
peroxiredoxin 2 family protein
geranyltranstransferase
short–chain type dehydrogenase_reductase
cytochrome P–450_1
virB4
virB2
virB3
virB6
virA
virB1
transposase_2
transposase
transposase_1
trag family protein
virB10_virB11
two–component response regulator
hyp_3
virB7_virB8_virB9
type iv secretion system lipoprotein 7
aldehyde dehydrogenase
family transcriptional regulator
msi151
rlxS
dithiobiotin synthetase
pyruvate dehydrogenase E1 component–like protein
ferredoxin
3–methyl–2–oxobutanoate hydroxymethyltransferase
abc–type multidrug transport atpase and permease component
noeJ
dihydrodipicolinate synthetase
hyp_9
succinoglycan biosynthesis regulator (exsB)
hypothetical protein_16
6–pyruvoyl tetrahydrobiopterin synthase
hyp_10
hypothetical protein_17
hyp_8
hyp_15
msi107
mosc domain–containing protein
trbJ
transcriptional regulator_3
hypothetical protein_15
serine hydroxymethyltransferase
S–adenosylmethionine synthetase
msi150
isoquinoline 1–oxidoreductase
nOLO
hyp_7
nodZ
phosphomannomutase
nucleoside–diphosphate–sugar epimerase
beta–ketoacyl–ACP synthase
hyp_6
hyp_4
type i secretion membrane fusion family
traF
nodD
nodD_1
transcriptional regulator
transcriptional regulator_1
hyp_5
traI1
hypothetical protein_7
Fis family transcriptional regulator
type i secretion membrane fusion family_1
aminotransferase
virB5
nodS
nodF
nodI
(4Fe–4S) ferredoxin_2
acyltransferase
msi110
nifB
fixX
hypothetical protein_10
nitrogenase stabilizing_protective protein
hypothetical protein_9
hemin–degrading family protein_1
hemin–degrading family protein
hypothetical protein_11
nodJ
fixC
N–acetylglucosaminyltransferase
cytochrome P450_1
nifS
homocitrate synthase
homocitrate synthase_1
hypothetical protein_13
cytochrome C peroxidase
hypothetical protein_12
hypothetical protein_14
hypothetical protein_8
transcriptional regulator_2
fixA
nodB
L–aspartate oxidase
fixB
Near-core symbiosis genes
Pairwise sequence identity for all orthologs in population
*
% Seq. ID
70
75
80
85
90
95
100
Cluster #
21
20
3
1
2
6
4
5
7
8
9
10
18
19
17
13
14
16
15
11
12
